# Novel 8-trifluoromethylquinobenzothiazines—Synthesis and Evaluation for Antiproliferative and Antibacterial Activity

**DOI:** 10.3390/ph19030422

**Published:** 2026-03-04

**Authors:** Daria Klimoszek, Anna Majewska, Małgorzata Jeleń, Marta Struga, Beata Morak-Młodawska, Małgorzata Dołowy

**Affiliations:** 1 Department of Analytical Chemistry, Faculty of Pharmaceutical Sciences in Sosnowiec, Medical University of Silesia in Katowice, Jagiellońska Street 4, 41-200 Sosnowiec, Poland; d201204@365.sum.edu.pl (D.K.); mdolowy@sum.edu.pl (M.D.); 2 Chair and Department of Medical Microbiology, Medical University of Warsaw, Chalubinski 5 Street, 02-004 Warsaw, Poland; anna.majewska@wum.edu.pl; 3 Department of Organic Chemistry, Faculty of Pharmaceutical Sciences, The Medical University of Silesia, Jagiellońska 4, 41-200 Sosnowiec, Poland; bmlodawska@sum.edu.pl; 4 Chair and Department of Biochemistry, Medical University of Warsaw, 02-097 Warsaw, Poland; marta.struga@wum.edu.pl

**Keywords:** azaphenothiazine, fluphenazine, triflupromazine, trifluoperazine, quinobenzothiazine, anticancer activity, antibacterial activity

## Abstract

**Background**: Phenothiazine derivatives bearing trifluoromethyl substituents have attracted increasing interest as multifunctional scaffolds in drug repositioning strategies, particularly in cancer and infectious diseases. Structural modification of classical phenothiazines by incorporation of a quinoline moiety has previously been shown to enhance biological activity. **Objectives**: The present study aimed to develop an efficient synthesis of 8-trifluoromethylquinobenzothiazines and to evaluate the anticancer and antibacterial potential of their N-substituted analogues inspired by triflupromazine, trifluoperazine, and fluphenazine. **Methods**: 6*H*-8-Trifluoromethylquinobenzothiazine was synthesized by cyclization of 2-amino-4-trifluoromethylbenzenethiol and 3-bromo-2-chloroquinoline. The resulting quinobenzothiazine, unsubstituted at the nitrogen atom, was subjected to N-alkylation reactions to afford eleven new 6-dialkylaminoalkyl derivatives. Structural elucidation was performed using NMR and HRMS techniques. Anticancer activity was evaluated by MTT assay against human breast (MDA-MB-231), pancreatic (Mia-PaCa-2), and lung (A-549) carcinoma cell lines, as well as normal HaCaT keratinocytes. Antibacterial activity was assessed by MIC/MBC determination against selected Gram-positive and Gram-negative reference strains and clinical isolates. **Results**: Among the synthesized compounds, derivatives **8** and **12** exhibited the most favorable anticancer profiles, showing micromolar cytotoxicity (IC_50_ ≈ 4–10 µM) against lung and pancreatic cancer cells combined with moderate selectivity toward cancer cells over normal keratinocytes. Compound **6** displayed lower cytotoxic potency but a notably high selectivity index due to minimal toxicity toward normal cells. In antibacterial assays, compound **3** exhibited activity against Gram-positive bacteria, including a methicillin-resistant *Staphylococcus aureus* isolate, with MIC values ranging from 7.8 to 15.6 µg/mL. The corresponding MBC values were equal to or twofold higher than the MICs (MBC/MIC = 1–2), fulfilling commonly accepted criteria for bactericidal activity (MBC/MIC ≤ 4). OD-based growth kinetics confirmed concentration-dependent inhibition of *S. aureus* growth. **Conclusions**: The obtained results identify 8-trifluoromethylquinobenzothiazines as a promising class of multifunctional compounds. Selected derivatives combine anticancer activity with acceptable selectivity or display potent antibacterial effects against clinically relevant Gram-positive pathogens.

## 1. Introduction

Cancer and bacterial infections remain leading global health challenges. According to the World Health Organization, cancer accounts for nearly 10 million deaths annually, with lung and pancreatic cancers ranking among the most lethal malignancies worldwide. Data from GLOBOCAN indicate a continuous increase in cancer incidence, emphasizing the urgent need for novel therapeutics with improved selectivity profiles [1]. Simultaneously, antimicrobial resistance (AMR) constitutes a major threat to global public health. Methicillin-resistant *Staphylococcus aureus* (MRSA) is classified among high-priority pathogens due to limited treatment options and high mortality rates [2].

Phenothiazine (PTZ) is a tricyclic heterocyclic compound containing nitrogen and sulfur atoms, first described by Heinrich A. Bernthesen at the end of the 19th century. Compounds containing a phenothiazine skeleton, initially used as dyes (e.g., methylene blue), quickly found applications in medicine, including in the treatment of malaria and as antiseptics [3,4,5]. In the mid-20th century, phenothiazines became the basis of modern psychiatry—chlorpromazine, the first antipsychotic drug, paved the way for the widespread use of PTZ as a privileged structure in pharmaceutical design [6]. At present, phenothiazines are a group of important compounds used not only in psychiatry, but also in the search for new therapeutics for cancer and bacterial diseases, including tuberculosis, as well as in materials chemistry as photoredox components [7,8,9].

They have become an important pillar of drug repositioning strategies due to their multidirectional effects on cancer cells and microorganisms, as well as their favorable pharmacological properties, which may accelerate clinical translation compared to the development of new molecular entities [10,11]. Their potential stems from their easy functionalization—various substitutions on benzene rings and nitrogen atoms allow for the modulation of both biological and physicochemical properties [7].

Compounds containing a trifluoromethyl group (–CF_3_) also occupy an important place within this class, as the CF_3_ moiety is widely used in drug design due to its ability to simultaneously modulate the electronic and lipophilic properties of molecules. Such properties translate into increased binding strength to the protein target, improved selectivity, and a favorable pharmacokinetic profile, including bioavailability and potential penetration into the CNS (central nervous system) [12,13,14,15]. In turn, pharmacological studies have shown that 2-CF_3_-PTZ derivatives may exhibit increased anticancer activity, including against oral cancer—in in vitro and in vivo studies, the efficacy of selected analogues was greater than that of classic phenothiazine drugs such as trifluoperazine [16]. In addition, synthetic reports indicate the possibility of obtaining stable and easily modifiable analogues of 2-CF_3_-PTZ, which opens up new perspectives in medicinal chemistry [15].

Triflupromazine, trifluoperazine, and fluphenazine—antipsychotic drugs containing a trifluoromethyl substituent at the second carbon of the phenothiazine ring—are increasingly becoming the subject of research into their potential repositioning because these drugs are multi-targeted, clinically well-known and often penetrate the CNS, which shortens the path to applications in new oncological and neurological indications and in infectious diseases. Their common theme is the modulation of cancer cell signaling pathways and the tumor microenvironment, as well as antibacterial activity, which may support therapies for resistant infections [10,11,17,18,19].

Triflupromazine, originally introduced as a sedative and antiemetic, effectively reduces anxiety and postoperative nausea while enhancing opioid and barbiturate effects without significantly affecting respiratory or hemodynamic function [20,21]. Although its clinical use has declined with the advent of second-generation neuroleptics, triflupromazine remains of scientific interest due to its diverse biological activity beyond central nervous system effects [22,23]. It exhibits potent antibacterial properties against numerous Gram-positive and Gram-negative strains, including *Staphylococcus aureus*, *Shigella* spp., and vibrios, with MIC values of 2–100 µg/mL, and increases survival in *Salmonella typhimurium*-infected mice [24]. Preclinical studies demonstrate that triflupromazine inhibits lactate dehydrogenase A (LDHA), suppressing glycolysis and enhancing cisplatin efficacy in lung cancer models, while also exerting cytotoxic effects on glioblastoma stem cells independent of dopamine receptor blockade [25]. Moreover, copper (II) complexes of triflupromazine with glycine or histidine display strong anticancer activity against liver (HepG2) and breast (MCF-7) cells, with low toxicity to normal cells [26].

Trifluoperazine (TFP) exhibits broad anticancer activity against various tumor types, including nasopharyngeal carcinoma (NPC) [27,28,29]. Thanks to its established safety profile and ability to cross the blood–brain barrier, trifluoperazine (TFP) represents a promising therapeutic option for central nervous system tumors and brain metastases [30,31]. In glioblastoma multiforme (GBM), TFP increases intracellular calcium levels by releasing Ca^2+^ from the endoplasmic reticulum through calmodulin (CaM) binding, thereby suppressing proliferation, migration, and invasion [30,31]. This mechanism also involves COX-2 upregulation, prostaglandin production, and PPARγ activation, leading to apoptosis in glioblastoma cells [32,33]. Additionally, TFP suppresses proliferation, migration, and clonogenicity of non-small cell lung cancer (NSCLC) cells, while its synthetic analog 3dc shows even stronger pro-apoptotic effects via caspase activation, DNA fragmentation, and inhibition of PI3K/AKT and ERK pathways. Both compounds significantly reduce tumor growth in vivo, supporting their potential in NSCLC therapy [34]. Furthermore, TFP exhibits neuroprotective effects against cytotoxic drug-induced neurotoxicity [35]. It may also modulate multidrug resistance by inhibiting calmodulin, P-glycoprotein, and topoisomerase II activity [36]. Finally, TFP demonstrates antiviral properties, effectively inhibiting dengue and Zika virus replication at the post-entry stage [37].

Fluphenazine, beyond its neuropsychiatric applications, exhibits therapeutic potential in oncology, infectious diseases, and as a tool for investigating drug resistance and membrane transport [38,39]. It shows cytotoxicity across multiple cancer cell types, including lung, breast, colon, liver, brain, leukemia, oral, ovarian, and skin cancers [38]. In triple-negative breast cancer (TNBC) models, fluphenazine inhibits proliferation, induces G0/G1 arrest, activates mitochondrial apoptosis, and suppresses migration and invasion by reducing ERK1/2 and AKT phosphorylation [40]. Molecular docking studies revealed that fluphenazine derivatives can inhibit caspase-3 by binding to its active site, forming stabilizing hydrogen bonds with Ser219 and Arg269 residues [41]. Phenothiazines, including fluphenazine, also modulate P-glycoprotein and signaling pathways while exhibiting antimicrobial, antioxidant, and neuroprotective effects [42]. In liver and breast cancer, fluphenazine induces cuproptosis—a copper-dependent cell death—and disrupts autophagy, limiting tumor growth [43,44]. It may also overcome drug resistance by sensitizing cells to antimitotic agents such as eribulin and inducing G2 arrest, DNA damage, and apoptosis via P-gp inhibition [45]. Additionally, fluphenazine shows antimicrobial activity against Gram-positive and Gram-negative bacteria, protecting mice from *S. typhimurium* infections as a “non-antibiotic” antimicrobial [46]. Structural optimization has yielded derivatives like CWHM-974 with enhanced antifungal potency and reduced resistance induction in C. albicans [39,47].

Our previous studies demonstrated that modifying the structure of phenothiazines by introducing one or two quinoline rings, leading to the formation of quinobenzothiazines and diquinothiazines, allowed for the creation of derivatives with multifaceted biological activity. Previous studies have demonstrated that selected quinoline-modified phenothiazines exhibit significant anticancer activity against numerous cancer cell lines, as well as antioxidant and immunomodulatory effects. These compounds inhibit mitogen-induced proliferation of peripheral blood mononuclear cells, limit TNFα production, and influence the activity of enzymes such as butyrylcholinesterase [48,49,50,51,52,53,54]. In vivo models, their anti-inflammatory and immunosuppressive effects have been observed, including inhibition of delayed-type hypersensitivity reactions, experimental psoriasis, and colitis, as well as an effect on the expression of IFNβ and genes dependent on this pathway [55,56]. The accumulated data indicate that phenothiazines modified with a quinoline ring constitute a promising class of compounds with potential use in the therapy of cancer and autoimmune diseases, which justifies their further modifications and studies on structure–activity relationships. 

**Figure 1:**
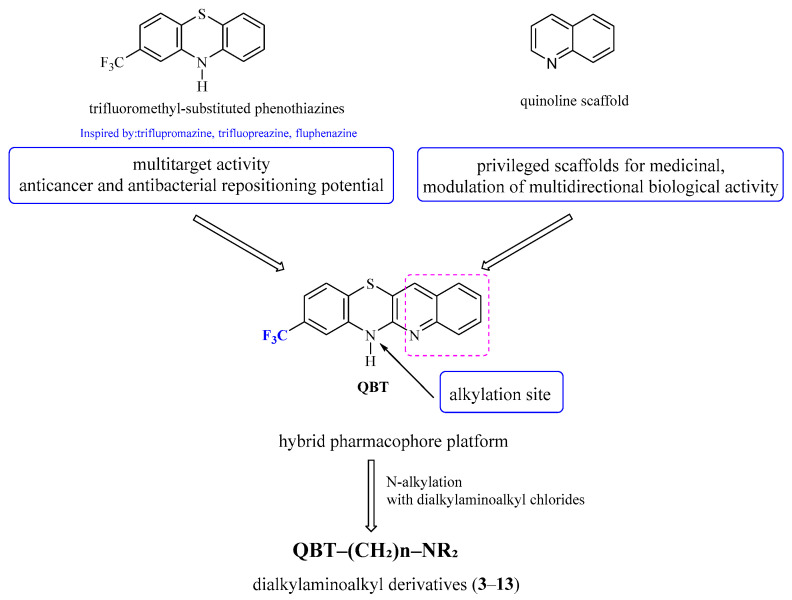
Rational hybrid design strategy of 8-trifluoromethylquinobenzothiazine derivatives. The scaffold combines structural elements of trifluoromethyl-substituted phenothiazines and a quinoline framework, followed by N-alkylation with dialkylaminoalkyl substituents inspired by clinically used phenothiazine derivatives.

Despite the well-documented biological activity of trifluoromethyl-substituted phenothiazines and their growing interest in drug repositioning strategies, structural modifications involving hybridization with quinoline frameworks remain insufficiently explored (Figure 1). In particular, quinobenzothiazine systems bearing a trifluoromethyl substituent have not been systematically investigated in the context of anticancer and antibacterial activity.

## 2. Results

### 2.1. Synthesis and Spectral Data

**Scheme 1:**
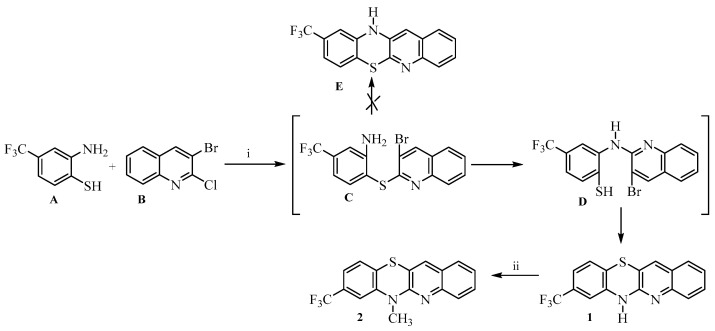
Reaction of 2-amino-4-trifluoromethylbenzenethiol **A** and 3-bromo-2-chloroquinoline **B** towards 8-trifluoromethylquinobenzothiazine **1**. Reagents and reaction conditions: (i) DMF, reflux, 1 h; (ii) DMF, NaH, r.t., 24 h.

In this study, a series of novel dialkylaminoalkyl derivatives of 8-trifluoromethylquinobenzothiazine were designed, synthesized, and evaluated for their anticancer and antibacterial activity. 6*H*-8-Trifluoromethylquinobenzothiazine **1** was prepared by the reaction of 2-amino-4-trifluoromethylbenzenethiol **A** and 3-bromo-2-chloroquinoline **B** (Scheme 1). The reaction was carried out in DMF under reflux for 1 h.

**Scheme 2:**
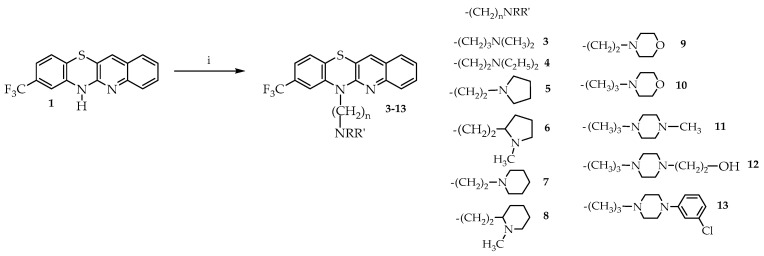
Synthesis of 6-dialkylaminoalkyl-8-(trifluoromethyl)-quinobenzothiazines **3**–**13**. Reagents and reaction conditions: (i) dioxane, NaOH, Cl(CH_2_)_n_R,R′.

We then carried out alkylation reactions with methyl iodide towards derivative 2 (Scheme 1), and with dialkylaminoalkyl chloride hydrochlorides towards the target derivatives **3**–**13** (Scheme 2).

### 2.2. Biological Evaluationof Newly Synthesized Compounds

#### 2.2.1. Cytotoxic Activity

The cytotoxic potential of newly synthesized compounds **1**–**13** (Scheme 1 and Scheme 2) was evaluated for their possible application in cancer therapy. Their effects were assessed on three human carcinoma cell lines: breast adenocarcinoma (MDA-MB-231), pancreatic cancer (Mia-PaCa-2), lung carcinoma (A-549), as well as on normal HaCaT keratinocytes, with IC_50_ values determined using the MTT assay [57,58]. For reference, the results were compared to the IC_50_ of the widely used chemotherapeutic agents doxorubicin and cisplatin (Table 1).

#### 2.2.2. In Vitro Antibacterial Activity

Next, the antibacterial efficacy of the newly synthesized 8-trifluoromethylquinobenzothiazines **1**–**13** was assessed by pre-screening them for minimum inhibitory concentrations (MICs). Most of the tested 8-trifluoromethylquinobenzothiazines inhibited the growth of bacteria used in the study at a concentration of ≥125 µg/mL. The most promising substances from the presented series were substances **3**, **5**, and **8** (Table 2).

The antibacterial activity of the investigated derivatives was evaluated against reference Gram-positive bacteria, including several *Staphylococcus aureus* strains (ATCC 6538, NCTC 4163, 2273 [MSSA], and T5591 [MRSA]) and *Enterococcus faecalis* (ATCC 29212), as well as against Gram-negative bacilli such as *Escherichia coli* (ATCC 25922) and *Pseudomonas aeruginosa* (ATCC 15442). All strains are susceptible to ciprofloxacin, a fluorophinolone antibiotic.

**Figure 2:**
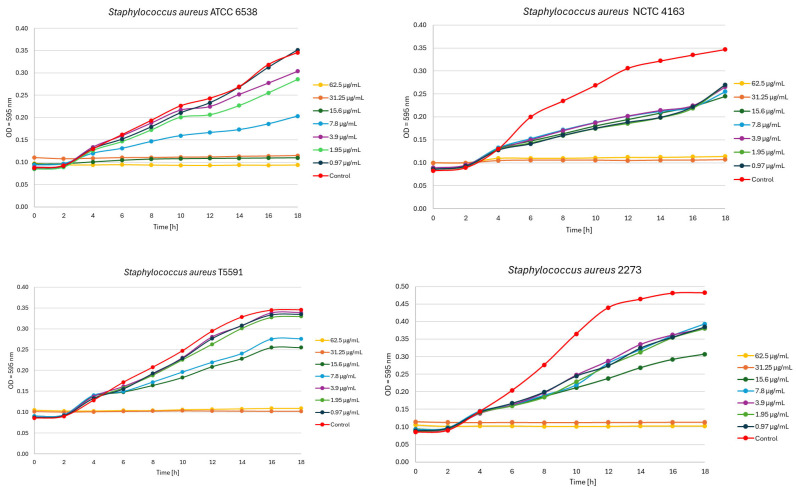
OD-based growth kinetics of *S. aureus* strains following exposure to compound **3**.

An OD-based growth kinetics study was also performed for the most active compound **3**. Figure 2 shows the growth inhibition of S. aureus strains based on OD exposed to compound **3**.

## 3. Discussion

The present study is a continuation of our ongoing efforts focused on the synthesis and biological evaluation of quinobenzothiazine derivatives [48]. Here, we aimed to develop an efficient and selective synthetic route to 6*H*-8-trifluoromethylquinobenzothiazine as a versatile core scaffold enabling the preparation of novel analogues of clinically established phenothiazines, namely triflupromazine, trifluoperazine, and fluphenazine. In the newly designed structures, the benzene ring of the phenothiazine system was replaced with a quinoline moiety, while diverse dialkylaminoalkyl substituents were introduced at the thiazine nitrogen atom (Figure 1). This structural modification was motivated by previously reported in vitro and in vivo activities of quinoline-modified phenothiazines, as well as by the well-documented anticancer and antibacterial potential of trifluoromethyl-substituted phenothiazine drugs [48]. The applied design strategy aimed to combine the favorable pharmacological features of trifluoromethyl phenothiazines with the biological relevance of the quinoline scaffold. The study was initiated by developing an efficient synthetic route to 6*H*-8-trifluoromethylquinobenzothiazine **1**. We used 2-amino-4-trifluoromethylbenzenethiol **A** and 3-bromo-2-chloroquinoline **B** as substrates (Scheme 1). The intermediate product in this type of syntheses of the phenothiazine system is phenylquinoline sulfide **C**. The initially formed phenylquinoline sulfide intermediate **C** may undergo Ullmann-type cyclization toward quinobenzothiazine **E** or proceed via a Smiles rearrangement (S→N type), in which the quinolinyl moiety migrates from sulfur to nitrogen, forming intermediate **D** that subsequently cyclizes to quinobenzothiazine **1**. According to literature data, the reaction progress of this type of the synthesis of phenothiazine systems in most cases depends on the conditions used [59,60]. If the products of Ullmann cyclization and Smiles rearrangement are the same, it is impossible to determine which path will lead to the closure of the phenothiazine system (this occurs when substrates not substituted with additional substituents are used). When the substrate in the synthesis of the quinobenzothiazine system is substituted o-aminobenzenethiol as a substrate, as in the reaction we performed, the possibility of formation of two phenothiazines **1** and **E** should be considered. We had already synthesized 6*H*-8-trifluoroquinobenzothiazine **1** in our earlier studies, but in those studies we used 2,2′-dichloro-3,3′-diquinolyl disulfide and *m*-trifluoromethylaniline [61]. This reaction resulted in the formation of 6*H*-8-trifluoromethylquinobenzothiazine and 6*H*-10-trifluoromethylquinobenzothiazine in yields of 28% and 22%, respectively, which required chromatographic separation. The separation was hampered by the small difference in R_f_ coefficients. In further studies, we confirmed the formation of the 8-trifluoromethylquinobenzothiazine system using single-crystal X-ray analysis [62]. Comparison of the substances obtained in these reactions allowed us to tentatively assume that the reaction of 2-amino-4-trifluoromethylbenzenethiol **A** and 3-bromo-2-chloroquinoline **B** involves a Smiles rearrangement. We unequivocally confirmed the assumed structure of the quinobenzothiazine system by measuring ^1^H NMR spectra and two-dimensional NOESY and COSY spectra (Table S1) of the 6-methyl derivative **2**. ^1^H NMR spectra also identified the product as 8-substituted quinobenzothiazine **2**. The proton signals of the quinoline moiety were observed at low field (above 7.2 ppm) and appeared as a doublet with an ortho coupling constant (proton H-4), a singlet (proton H-12), a triplet with two ortho couplings (proton H-2), and a multiplet consisting of the signals from protons H-1 and H-3. The proton signals of the benzene ring were observed at high field (below 7.23 ppm) and were observed as two signals with a shape and multiplicity depending on the proton to which they were assigned. The proton signals H-10 and H-9 appeared as one multiplet with a multiplicity of 2, while the proton signal H-7 appeared as a broad singlet. To fully confirm the structure of the obtained quinobenzothiazine system, a ^13^C NMR spectrum and two-dimensional HSQC and HMBC spectra were also obtained. These spectra allowed the assignment of the appropriate C atoms to the individual signals. As a result, the products were identified as 8-trifluoromethylquino[3,2-b]benzo[1,4]thiazine (8-trifluoromethylbenzo[b]-1-azaphenothiazine) (Table S2).

Moreover, characteristic splitting signals originating from the CF_3_ group were observed in the ^13^C NMR spectra, as expected. The CF_3_ carbon gave signals at a shift of approximately 122.7–124.8 ppm, depending on the derivative, as quartets. The coupling constants for these signals were in the range ^1^*J*_CF_ = 214–279 Hz. Similarly, the C-8 carbon with a CF_3_ substituent in the benzene ring was observed as quartets with δ from 129.5 to 130.9, depending on the derivative. The coupling constants for these signals were in the range ^2^*J*_CF_ = 29.7–34.5 Hz. This effect of the CF_3_ substituent on the ^13^C NMR spectrum is also consistent with previously published data for compounds containing a CF_3_ substituent [63].

The use of 2-amino-4-trifluoromethylbenzenethiol **A** and 3-bromo-2-chloroquinoline **B** as starting materials allowed for the selective and efficient synthesis of 6*H*-8-trifluoromethylquinobenzothiazine, which was subsequently used to obtain derivatives containing N,N-dialkylaminoalkyl substituents at the 6-position, including the quinoline analogs triflupromazine, trifluoperazine, and fluphenazine. N,N-dialkylaminoalkyl derivatives were obtained by N-alkylation reactions of selected acyclic and cyclic dialkylaminoalkyl chloride hydrochlorides. The reactions were carried out in refluxing 1,4-dioxane in the presence of sodium hydroxide. These syntheses allowed for the preparation of eleven different 6-dialkylaminoalkyl derivatives **3**–**13** with good efficiency (Scheme 2).

Next, the cytotoxic potential of newly synthesized triflupromazine, trifluoperazine and fluphenazine derivatives **3**–**13** (Scheme 2) and compounds **1** and **2** (Scheme 1) was evaluated against three cancer cell lines: breast adenocarcinoma (MDA-MB-231), pancreatic cancer (Mia-PaCa-2), lung cancer (A-549), as well as on normal HaCaT keratinocytes (Table 1). Among the thirteen new, tested 6-substituted 8-trifluoromethylquinobenzothiazines **1**–**13**, three derivatives—**8** (with N-methylpiperidine ethyl substituent), **12** (with hydroxyethylpiperazinylpropyl substituent), and **6** (with N-methylpyrrolidinoethyl substituent)—were distinguished by favorable cytotoxicity and selectivity profiles, justifying further investigation of their anticancer potential. Compound **8** demonstrated uniform and moderate activity against three cancer cell lines: MDA-MB-231 (human breast adenocarcinoma), Mia-PaCa-2 (human pancreatic cancer), and A-549 (human lung carcinoma), achieving IC_50_ values in the range of 6.98–9.96 µM with relatively low toxicity to normal HaCaT keratinocytes (13.06 µM), which translates into a selectivity index (SI) in the range of 1.3–1.9. This combination of moderate potency and advantage over cancer cells makes compound **8** a particularly interesting candidate for further development. From a translational perspective, the relevance of this activity is strengthened by prior evidence demonstrating that ER-negative breast cancer cells can be effectively targeted by phenothiazine-derived agents. In a study conducted by Jeannine S. Strobl et al. [64], thioridazine markedly suppressed the proliferation of the triple-negative breast cancer cell line MDA-MB-231 following 72 h of exposure, demonstrating antiproliferative efficacy comparable to that of pimozide. Importantly, the authors proposed thioridazine as a potential non-cytotoxic alternative to tamoxifen for tamoxifen-resistant breast cancer. Thioridazine elicited also dose-dependent cytotoxicity in A549 sphere cells (lung cancer stem-like cells), inducing caspase-dependent apoptosis and G0/G1 cell cycle arrest, with significant inhibitory effects on xenografts in mice [65]. Guanhua Qian et al. [66] demonstrated that thioridazine induced cell death in A549 cells and enhanced cisplatin sensitivity through mitochondria-dependent apoptosis, with the combination treatment producing the highest percentage of dead cells [66]. Compound **12** showed the highest activity against A-549 cells (IC_50_ = 4.22 µM) and Mia-PaCa-2 (IC_50_ = 5.16 µM), whereas its activity against MDA-MB-231 was weaker (IC_50_ = 10.76 µM), suggesting a relative dependence of activity on tumor type. Its activity toward MDA-MB-231 is consistent with previous observations for fluphenazine, which inhibits proliferation, migration, and invasion of this cell line, although its efficacy varies depending on cancer type. The enhanced activity of compound 12 in lung and pancreatic cancer cells may indicate the involvement of molecular targets particularly relevant in these tumor types [67]. Quinobenzothiazine **6**, although it had significantly higher IC_50_ values (approx. 43–48 µM for Mia-PaCa-2 and A-549), was non-toxic to HaCaT (IC_50_ > 100 µM), resulting in SI values exceeding 2. Such a wide therapeutic window, despite its lower potency, makes this compound an interesting scaffold for future structural modifications aimed at enhancing its anticancer activity while maintaining high selectivity. A comparative analysis of selectivity indices further highlights the distinct profiles of compounds **8** and **12** relative to the reference drug doxorubicin. Doxorubicin exhibited submicromolar IC_50_ values against MDA-MB-231 (0.5 µM) and A-549 (0.26 µM), confirming its high intrinsic potency. However, its cytotoxicity toward normal HaCaT keratinocytes (IC_50_ = 0.3 µM) resulted in low selectivity indices (SI = 0.60 for MDA-MB-231 and SI = 1.15 for A-549), indicating a narrow therapeutic margin. Notably, in the Mia-PaCa-2 model, doxorubicin showed markedly reduced activity (IC_50_ = 24.2 µM) combined with extremely poor selectivity (SI = 0.01), reflecting comparable or even greater toxicity toward normal cells than pancreatic cancer cells in this experimental setting. In contrast, compound 8 achieved SI values of 1.31–1.87, while compound **12** reached SI values of 1.40 (Mia-PaCa-2) and 1.71 (A-549), exceeding that of doxorubicin in the lung carcinoma model. Although their absolute potency was lower, both compounds demonstrated a more advantageous therapeutic window. Compound **8**, containing a substituted piperidine ring, and compound **12**, incorporating a piperazine derivative bearing a hydroxyethyl fragment, demonstrated higher cytotoxic potency than simple aliphatic analogues. Cyclic amines introduce conformational restriction and defined spatial orientation of the nitrogen atom, which may favor binding to protein targets involved in proliferation pathways. In contrast, more flexible side chains may lead to reduced target specificity. An especially interesting case is compound **6**, which displayed moderate antiproliferative potency but minimal toxicity toward normal cells (IC_50_ > 100 µM). Consequently, the calculated selectivity indices were SI > 2.30 for Mia-PaCa-2 and SI > 2.09 for A-549, representing the highest selectivity observed within the series. Although compound **6** is less potent than doxorubicin in terms of absolute IC_50_ values, its markedly reduced cytotoxicity toward non-malignant cells suggests a substantially improved safety margin and identifies this scaffold as a promising candidate for further structural optimization. Derivative **11** (with methylpiperazinylpropyl substituent), a quinoline analogue of trifluoperazine, was characterized by an IC_50_ in the range of 11.31–17.88 µM against cancer cells, but its toxicity towards normal cells was comparable (IC_50_ = 11.29 µM), resulting in an SI close to 1 or <1. This indicates no preferential treatment for transformed cells. Derivatives **5** (with a pyrrolidinoethyl substituent) and **7** (with a piperidinoethyl substituent), which do not contain additional substituents in the cyclic amine fragment, showed moderate cytotoxic activity against all three cancer lines (IC_50_~19–50 µM), but their selectivity towards normal cells was low (SI in the range of 0.4–1.3). This means that their activity does not significantly differentiate between transformed and normal cells, which significantly reduces the translational potential of these compounds.

Introduction of morpholino substituents (derivatives **9** and **10**) abolished anticancer activity while retaining cytotoxicity toward normal cells. No significant differences were observed with respect to the linker length between the thiazine and morpholine rings.

A direct comparison with the parent scaffold clearly demonstrates that N-alkylation is essential for biological activity. The unsubstituted 6*H*-8-trifluoromethylquinobenzothiazine **1** and its simple N-methyl derivative **2** exhibited negligible cytotoxic and antibacterial effects, whereas introduction of dialkylaminoalkyl substituents led to a substantial increase in activity. This observation confirms that the quinobenzothiazine core alone does not constitute a fully active pharmacophore and requires appropriate side-chain functionalization to exert biological effects.

In studies of the antibacterial activity of newly synthesized derivatives, molecule **3** containing a dimethylaminopropyl moiety was the only compound tested to exhibit antibacterial activity within the concentration range assessed. Quinobenzothiazine **3** inhibited the growth of *S. aureus* reference strains and clinical isolates with MIC values of 7.8–15.6 µg/mL. The corresponding MBC values were equal to the MIC or 2× MIC. Compound **3** also showed activity against *E. faecalis* ATCC 29212. In contrast, its activity against Gram-negative bacteria was limited: for *E. coli* ATCC 25922 the MIC and MBC were 125 µg/mL (MBC/MIC = 1), whereas no inhibitory effect against *P. aeruginosa* ATCC 15442 was observed at the highest concentration tested (MIC > 250 µg/mL). Overall, the MBC/MIC ratios for compound **3** ranged from 1 to 2 for all strains where MBC could be determined, consistent with a bactericidal effect under the test conditions. OD-based growth kinetics demonstrated a clear, concentration-dependent inhibition of *S. aureus* following exposure to compound **3** (0.97–62.5 µg/mL), with optical density measurements recorded every 2 h. For all strains increasing compound **3** concentrations progressively reduced the OD increase compared with the growth control (inoculated medium without the compound). At low quinobenzothiazine 3 concentrations (0.97–3.9 µg/mL), the growth curve of *S. aureus* ATCC 6538 and T5591 remained comparable to the growth control, whereas the remaining staphylococcal strains exhibited a pronounced shift of the growth curve relative to the control and a reduction in maximum optical density, indicating a higher susceptibility of these strains to the tested compound. This pattern reflects a sub-inhibitory effect characterized by impaired growth kinetics, while the bacterial population retained the capacity for continued proliferation. At the highest **3** concentrations (31.25–62.5 µg/mL), OD trajectories remained close to baseline throughout the monitoring period, indicating strong suppression of bacterial growth. Figure 2 presents OD-based growth inhibition of *S. aureus* strains exposed to compound **3**.

*S. aureus* is a major cause of severe multiorgan infections, and methicillin-resistant strains (MRSA) rank among the leading bacterial contributors to mortality attributable to antimicrobial resistance worldwide [68]. Quinobenzothiazine 3 demonstrated a clear preference for Gram-positive bacteria, as reflected by low MIC values against both reference and clinical *S. aureus* strains, including an MRSA isolate expressing an inducible MLSB phenotype (iMLSB), as well as comparable activity against *E. faecalis*. The corresponding MBC/MIC ratios (1–2) indicate that concentrations required to inhibit growth were bactericidal under in vitro conditions. In contrast, compound 3 showed reduced activity against *E. coli* and no measurable inhibitory effect against *P. aeruginosa* within the tested concentration range. Against the backdrop of the global burden of bacterial infections and the limitations of currently available therapies, there remains a clear need for continued discovery of novel antibacterial compounds and chemical optimization of promising lead structures to improve efficacy across a range of clinically relevant pathogens.

## 4. Materials and Methods

### 4.1. Chemical Part

Melting points were measured in open capillary tubes using a Boetius melting point apparatus (Stuart Equipment, Stone, UK) and are reported without correction. NMR spectra were acquired on Bruker Avance instruments (Bruker, Billerica, MA, USA), operating at 600 MHz for ^1^H, 150 MHz for ^13^C and 564 MHz for ^19^F nuclei in CDCl_3_ as solvents. For selected compounds, two-dimensional NMR experiments (COSY, NOESY, HSQC, and HMBC) were performed at 600 MHz employing the COSYGPSW, NOESYGPPHSW, HSQCGPPH, and HMBCGP pulse sequences. High-resolution mass spectra (HRMS) were obtained using electron impact (EI) ionization on a Bruker Impact II mass spectrometer (Bruker, Billerica, MA, USA). The ^1^H NMR, ^13^C NMR, and HRMS spectra are provided in the Supplementary Materials. Thin-layer chromatography (TLC) analyses were carried out on neutral aluminum oxide 60 F_254_ plates (type E; Merck 1.05581, Darmstadt, Germany) with dichloromethane (CH_2_Cl_2_) as the eluent.

General procedure for the synthesis of 8-trifluoromethylquinobenzothiazine derivatives:

- Synthesis of 6H-8-trifluoromethylquinobenzothiazine **1**.

A solution of 0.24 g (1 mmol) of 3-bromo-2-chloroquinoline **B** in 10 mL of dry DMF was prepared and heated to reflux, followed by the addition of 0.16 g (1 mmol) of 2-amino-4-trifluoromethylthiophenol **A**. The mixture was heated at reflux for 1 h. It was then cooled and poured into 30 mL of water. The precipitate was filtered off, washed with water, dried, and purified by crystallization from ethanol to give 6*H*-8-trifluoromethylquinobenzothiazine **1**:

Yield: 88%. M.p.: 220–221 °C. ^1^H NMR (CDCl_3_) δ: 7.00 (s, 1H, H-7), 7.06–7.07 (d, 1H, H-10, *J* = 6.5 Hz), 7.16–7.18 (d, 1H, H-9, *J* = 8 Hz), 7.37 (t, 1H, H-2, *J* = 6.5 Hz), 7.53 (d, 1H, H-1, *J* = 6.5 Hz), 7.57 (t, 1H, H-3, *J* = 6.5 Hz), 7.63 (s, 1H, H-12), 7.64 (d, 1H, H-4, *J* = 8.5 Hz). ^13^C NMR (150 MHz, CDCl_3_) δ: 112.68, 115.67, 120.29, 121.18, 123.40, 124.42, 125.34, 125.99, 126.33, 126.70, 130.24, 130.64, 132.79, 137.56, 142.80, 149.53. HRMS (ESI) calcd for C_16_H_10_F_3_N_2_S [M+H]^+^: 319.0517, found: 319.0519.

- Synthesis of 6-methyl-8-trifluoromethyl-quinobenzothiazine **2**.

A suspension of 0.16 g (0.5 mmol) of 6*H*-8-trifluoromethylquinobenzothiazine **1** and 0.06 g of NaH (2.5 mmol) in 5 mL of dry DMF was prepared and stirred at room temperature for 0.5 h. After this time, 0.05 mL of methyl iodide (0.75 mmol) was added and stirring was continued for 24 h. The reaction mixture was then poured into 30 mL of water. The resulting precipitate was filtered off, washed with water, and purified by column chromatography (alumina, CH_2_Cl_2_) to give 6-methyl-8-trifluoromethylquinobenzothiazine **2**:

Yield: 76%. M.p.: 115–116 °C. ^1^H NMR (CDCl_3_) δ: 3.65 (s, 3H, CH_3_), 7.10 (s, 1H, H-7), 7.19–7.23 (m, 2H, H-9, H-10), 7.32 (t, 1H, H-2, *J* = 6 Hz), 7.54–7.57 (m, 2H, H-1, H-3), 7.69 (s, 1H, H-12), 7.81 (d, 1H, H-4, *J* = 6 Hz). ^13^C NMR (150 MHz, CDCl_3_) δ: 33.87, 111.81 (C-7, ^3^*J* = 4.5 Hz), 117.46 (C-10a), 119.30 (C-9, ^3^*J* = 4.5 Hz), 123.99 (CF_3_, ^1^*J* = 271.5 Hz), 124.65 (C-2), 125.35 (C-5a), 125.98 (C-4a), 126.33 (C-3), 126.72 (C-10), 127.48 (C-4), 129.49 (C-1), 130.03 (C-8, ^2^*J* = 31.5 Hz), 132.40 (C-12), 143.37 (C-6a), 145.74 (C-12a), 152.48 (C-11a). HRMS (ESI) calcd for C_17_H_12_F_3_N_2_S [M+H]^+^: 333.0673, found: 333.0682.

- Synthesis of 6-dialkylaminoalkyl-8-trifluoromethylquinobenzothiazines **3**–**13**.

A mixture of 6*H*-8-trifluoroquinobenzothiazine **1** (0.16 g, 0.5 mmol) and sodium hydroxide (0.30 g, 7.5 mmol) in 5 mL of dioxane was heated at reflux for one hour. Then, dialkylaminoalkyl chloride hydrochloride (1.5 mmol, 3-dimethylaminopropyl—0.24 g,2-diethylaminoethyl—0.26 g, 2-(1-pyrrolidyl)ethyl—0.26 g, 2-(1-methylpyrrolidin-2-yl)ethyl—0.27 g, 2-(1-piperidyl)ethyl—0.26 g, 2-(1-methyl-2-piperidinyl)ethyl—0.30 g, 2-(1-morpholinyl)ethyl—0.28 g, 3-(1-morpholinyl)propyl—0.30 g, 3-(4-methylpiperazin-1-yl)propyl 0.38 g, 3-(4-hydroxyethylpiperazin-1-yl)propyl 0.42 g, 4-(3-chlorophenyl)piperazin-1-yl)propyl—0.47 g) was added to the reaction system and heating at reflux was continued for 3 h. Then the reaction mixture was cooled and poured into water (25 mL). The resulting solution was extracted with chloroform. The combined extracts were washed with water to pH = 7 and dried over Na_2_SO_4_. The chloroform was evaporated in vacuo, and the residue was purified by column chromatography (Al_2_O_3_, CHCl_3_) to afford compounds **3**–**13**:

N,N-dimethyl-3-(8-(trifluoromethyl)-quino[3,2-b]benzo[1,4]thiazin-6-yl)propan-1-amine (**3**):

Yield: 65%. M.p.: 75–77 °C. ^1^H NMR (CDCl_3_) δ: 2.07–2.09 (m, 2H, CH_2_), 2.36 (s, 6H, 2CH_3_), 2.54–2.57 (m, 2H, CH_2_), 4.25–4.27 (m, 2H, CH_2_), 7.10–7.11 (m, 2H, H-7, H-9), 7.17 (s, 1H, H-10), 7.26 (t, 1H, H-2, *J* = 6 Hz), 7.46–7.51 (m, 2H, H-1, H-3), 7.54 (s, 1H, H-12), 7.70 (d, 1H, H-4, *J* = 6 Hz). ^13^C NMR (150 MHz, CDCl_3_) δ: 23.89, 43.93, 45.26, 57.20, 112.03 (^3^*J* = 3.0 Hz), 117.26, 119.07 (^3^*J* = 3.0 Hz), 124.53 (^1^*J* = 273.0 Hz), 125.06, 126.07, 126.20, 126.62, 126.85, 127.48, 129.38, 129.91 (^2^*J* = 31.3 Hz), 132.01, 141.96, 145.73, 151.40. ^19^F NMR (564 MHz, CDCl_3_) δ: −62.83. HRMS (ESI) calcd for: C_21_H_21_F_3_N_3_S [M+H]^+^: 404.1408, found: 404.1401.

N,N-diethyl-2-(8-(trifluoromethyl)-quino[3,2-b]benzo[1,4]thiazin-6-yl)ethan-1-amine (**4**):

Yield: 71%. M.p.: Yellow oil. ^1^H NMR (CDCl_3_) δ: 1.18–1.25 (m, 6H, 2CH_3_), 2.76 (m, 4H, 2CH_2_), 2.94 (m, 2H, CH_2_), 4.33 (m, 2H, CH_2_), 6.98 (d, 1H, H-7), 7.05–7.08 (m, 2H, H-9, H-10), 7.21 (t, 1H, H-2, *J* = 6 Hz), 7.43–7.45 (m, 2H, H-1, H-3), 7.53 (s, 1H, H-12), 7.62 (d, 1H, H-4, *J* = 6 Hz). ^13^C NMR (150 MHz, CDCl_3_) δ: 11.47, 29.72, 47.05, 47.82, 112.23 (^3^*J* = 3.0 Hz), 117.06, 120.29 (^3^*J* = 3.0 Hz), 123.63 (^2^*J* = 279.0 Hz), 124.36, 126.13, 126.23, 126.68, 126.79, 127.38, 129.45, 129.93 (^1^*J* = 31.3 Hz), 131.95, 145.66, 149.68, 150.95. ^19^F NMR (564 MHz, CDCl_3_) δ: −62.88. HRMS (ESI) calcd for: C_22_H_23_F_3_N_3_S [M+H]^+^: 418.1565, found: 418.1574.

6-(2-(pyrrolidin-1-yl)ethyl)-8-(trifluoromethyl)-quino[3,2-b]benzo[1,4]thiazine (**5**):

Yield: 67% M.p.: Yellow oil. ^1^H NMR (CDCl_3_) δ: 2.17–2.20 (m, 4H, 2CH_2_), 3.50–3.53 (m, 4H, 2CH_2_), 3.86–3.88 (m, 2H, CH_2_), 4.76 (t, 2H, CH_2_, *J* = 7.2 Hz), 7.14 (d, 1H, H-7, *J* = 3.6 Hz), 7.19–7.21 (m, 2H, H-9, H-10), 7.28 (t, 1H, H-2, *J* = 7.2 Hz), 7.48–7.50 (m, 2H, H-1, H-3), 7.63–7.69 (m, 2H, H-4, H-12). ^13^C NMR (150 MHz, CDCl_3_) δ: 23.46, 41.45, 50.69, 53.70, 112.13 (^3^*J* = 3.3 Hz), 117.63, 120.30 (^3^*J* = 3.5 Hz), 124.25, 124.43 (^1^*J* = 214.0 Hz), 125.35, 126.21, 126.52, 127.21, 127.43, 129.86, 130.72 (^2^*J* = 32.7 Hz), 132.78, 141.35, 145.16, 150.78. ^19^F NMR (564 MHz, CDCl_3_) δ: −62.75. HRMS (ESI) calcd for: C_22_H_21_F_3_N_3_S [M+H]^+^: 416.1408, found: 416.1411.

6-(2-(1-methylpyrrolidin-2-yl)ethyl)-8-(trifluoromethyl)-quino[3,2-b]benzo[1,4]thiazine (**6**):

Yield: 71% M.p.: Yellow oil. ^1^H NMR (CDCl_3_) δ: 1.83–1.79 (m, 2H, CH_2_), 1.83–1.93 (m, 2H, CH_2_), 2.04–2.07 (m, 2H, CH_2_), 2.17–2.22 (m, 2H, CH_2_), 2.34 (s, 3H, CH_3_), 4.19 (t, 2H, CH_2_, *J* = 5.9 Hz), 7.04 (m, 3H, H-7, H-9, H-10), 7.18 (t, 1H, H-2, *J* = 3.8 Hz), 7.40–7.42 (m, 2H, H-1, H-3), 7.50 (s, 1H, H-12), 7.60 (d, 1H, H-4, *J* = 6 Hz). ^13^C NMR (150 MHz, CDCl_3_) δ: 22.11, 28.95, 30.19, 40.23, 42.86, 56.87, 65.02, 111.93 (^3^*J* = 3 Hz), 117.22, 119.07 (^3^*J* = 3.5 Hz), 123.54, 124.32 (^1^*J* = 233.7 Hz), 124.60, 126.07, 126.20, 126.89, 127.46, 129.39, 129.84 (^2^*J* = 32.6 Hz), 132.02, 141.83, 145.71, 151.31. ^19^F NMR (564 MHz, CDCl_3_) δ: −62.67. HRMS (ESI) calcd for: C_23_H_23_F_3_N_3_S [M+H]^+^: 430.1565, found: 430.1567.

6-(2-(piperidin-1-yl)ethyl)-8-(trifluoromethyl)-quino[3,2-b]benzo[1,4]thiazine (**7**):

Yield: 73%. M.p.: Yellow oil. ^1^H NMR (CDCl_3_) δ: 1.82–1.88 (m, 4H, 2CH_2_), 2.24–2.28 (m, 2H, CH_2_), 2.82–2.83 (m, 2H, CH_2_), 3.41 (m, 2H, CH_2_), 3.66–3.67 (m, 2H, CH_2_), 4.87 (t, 2H, CH_2_, *J* = 6.6 Hz), 7.20 (m, 3H, H-7, H-9, H-10), 7.30 (t, 1H, H-2, *J* = 7.8 Hz), 7.50–7.53 (m, 2H, H-1, H-3), 7.67 (s, 1H, H-12), 7.76 (d, 1H, H-4, *J* = 9 Hz). ^13^C NMR (150 MHz, CDCl_3_) δ: 23.36, 24.56, 29.71, 53.94, 54.47, 112.39, 117.29, 119.51, 124.01 (^1^*J* = 245.3 Hz), 124.85, 126.13, 126.29, 126.67, 126.95, 127.42, 129.52, 130.23 (^2^*J* = 32.3 Hz), 132.17, 141.72, 145.52, 150.96. ^19^F NMR (564 MHz, CDCl_3_) δ: −62.47. HRMS (ESI) calcd for: C_23_H_23_F_3_N_3_S [M+H]^+^: 430.1565, found: 430.1579.

6-(2-(1-methylpiperidin-2-yl)ethyl)-8-(trifluoromethyl)-quino[3,2-b]benzo[1,4]thiazine (**8**):

Yield: 68%. M.p.: 115–116 °C. ^1^H NMR (CDCl_3_) δ: 1.73–1.78 (m, 4H, 2CH_2_), 1.85–1.93 (m, 2H, CH_2_), 2.13–2.21 (m, 2H, CH_2_), 2.28–2.34 (m, 2H, CH_2_), 2.53 (s, 3H, CH_3_), 4.16–4.37 (m, 2H, CH_2_), 7.09 (s, 1H, H-7), 7.13 (s, 2H, H-9, H-10), 7.28 (t, 1H, H-2, *J* = 4.0 Hz), 7.49–7.52 (m, 2H, H-1, H-3), 7.57 (s, 1H, H-12), 7.68 (d, 1H, H-4, *J* = 8.6 Hz). ^13^C NMR (150 MHz, CDCl_3_) δ: 23.83, 25.24, 27.99, 30.43, 42.82, 56.79, 62.64, 111.57 (^3^*J* = 3.8 Hz), 117.00, 118.98 (^3^*J* = 3.5 Hz), 123.92 (^2^*J* = 246.6 Hz), 124.58, 124.84, 126.05, 126.18, 126.81, 127.36, 129.38, 129.30 (^2^*J* = 32.9 Hz), 131.84, 141.73, 145.74, 150.92. ^19^F NMR (564 MHz, CDCl_3_) δ: −62.75. HRMS (ESI) calcd for: C_24_H_25_F_3_N_3_S [M+H]^+^: 444.1721, found: 444.1727.

4-(3-(8-(trifluoromethyl)-quino[3,2-b]benzo[1,4]thiazin-6-yl)ethyl)morpholine (**9**):

Yield: 64%. M.p.: 105–106 °C. ^1^H NMR (CDCl_3_) δ: 2.74–2.95 (m, 8H, 4CH_2_), 3.80–3.91 (m, 2H, CH_2_), 4.38–4.51 (m, 2H, CH_2_), 6.89 (s, 1H, H-7), 7.08 (m, 2H, H-9, H-10), 7.22 (t, 1H, H-2, *J* = 7.2 Hz), 7.43–7.46 (m, 2H, H-1, H-3), 7.55 (s, 1H, H-12), 7.63 (d, 1H, H-4, *J* = 8.7 Hz). ^13^C NMR (150 MHz, CDCl_3_) δ: 29.71, 53.06, 53.62, 66.16, 112.48 (^3^*J* = 3.2 Hz), 117.29, 119.46 (^3^*J* = 3.3 Hz), 122.74 (^1^*J* = 247.8 Hz), 124.84, 126.13, 126.29, 126.75, 126.95, 127.42, 129.54, 130.09 (^2^*J* = 32.3 Hz), 132.21, 141.83, 145.55, 151.12. ^19^F NMR (564 MHz, CDCl_3_) δ: −62.50. HRMS (ESI) calcd for: C_22_H_21_F_3_N_3_OS [M+H]^+^: 432.1357, found: 432.1360.

4-(3-(8-(trifluoromethyl)-quino[3,2-b]benzo[1,4]thiazin-6-yl)propyl)morpholine (**10**):

Yield: 64%. M.p.: Yellow oil. ^1^H NMR (CDCl_3_) δ: 2.14 (m, 2H, CH_2_), 2.60 (m, 4H, 2CH_2_), 3.79–3.85 (m, 6H, 3CH_2_), 4.35 (m, 2H, CH_2_), 7.12 (s, 1H, H7), 7.15–7.18 (m, 2H, H-9, H-10), 7.30 (t, 1H, H-2, *J* = 7.1 Hz), 7.52–7.55 (m, 2H, H-1, H-3), 7.63 (s, 1H, H-12), 7.70 (d, 1H, H-4, *J* = 8.6 Hz). ^13^C NMR (150 MHz, CDCl_3_) δ: 43.61, 53.61, 56.31, 66.56, 111.98 (^3^*J* = 4.0 Hz), 117.37, 119.11, 119.13, 124.64, 124.75 (^1^*J* = 224.8 Hz), 126.09, 126.25, 126.93, 127.37, 129.46, 129.85 (^2^*J* = 29.7 Hz), 132.1013, 142.00, 145.70, 151.50. ^19^F NMR (564 MHz, CDCl_3_) δ: −62.67. HRMS (ESI) calcd for: C_23_H_23_F_3_N_3_OS [M+H]^+^: 446.1514, found: 446.1523.

6-(3-(4-methylpiperazin-1-yl)propyl)-8-(trifluoromethyl)-quino[3,2-b]benzo[1,4]thiazine (**11**):

Yield: 70%. M.p.: 108–109 °C. ^1^H NMR (CDCl_3_) δ: 2.06–2.09 (m, 2H, CH_2_), 2.35–2.38 (m, 5H, CH_2_, CH_3_), 2.51–2.55 (m, 2H, CH_2_), 2.60 (s, 4H, 2CH_2_), 3.61 (m, 2H, CH_2_), 4.31 (m, 2H, CH_2_), 7.12 (s, 1H, H7), 7.13–7.16 (m, 2H, H-9, H-10), 7.29 (t, 1H, H-2, *J* = 5.8 Hz), 7.51–7.54 (m, 2H, H-1, H-3), 7.61 (s, 1H, H-12), 7.69 (d, 1H, H-4, *J* = 8.7 Hz). ^13^C NMR (150 MHz, CDCl_3_) δ: 23.39, 43.72, 45.68, 52.79, 54.90, 55.69, 112.03 (^3^*J* = 3.5 Hz), 117.40, 119.01 (^3^*J* = 3.5 Hz), 124.00 (^1^*J* = 270.9 Hz), 125.23, 126.07, 126.21, 126.86, 127.42, 129.39, 129.78 (^2^*J* = 31.9 Hz), 129.99, 132.00, 142.07, 145.75, 151.54. ^19^F NMR (564 MHz, CDCl_3_) δ: −63.00. HRMS (ESI) calcd for: C_24_H_26_F_3_N_4_S [M+H]^+^: 459.1830, found: 459.1820.

2-(4-(3-(8-(trifluoromethyl)-quino[3,2-b]benzo[1,4]thiazin-6-yl)propyl)piperazin-1-yl)ethan-1-ol (**12**):

Yield: 67%. M.p.: Yellow oil. ^1^H NMR (CDCl_3_) δ: 1.96–1.98 (m, 2H, CH_2_), 2.06–2.10 (m, 2H, CH_2_), 2.51–2.54 (m, 4H, 2CH_2_), 2.60–2.62 (m, 3H, OH, CH_2_), 3.60–3.62 (m, 2H, 2CH_2_), 3.65–3.68 (m, 4H, 2CH_2_), 4.32 (s, 2H, CH_2_), 7.12 (s, 1H, H-7), 7.15–7.16 (m, 2H, H-9, H-10), 7.30 (t, 1H, H-2, *J* = 7.8 Hz), 7.52–7.54 (m, 2H, H-1, H-3), 7.63 (s, 1H, H-12), 7.70 (d, 1H, H-4, *J* = 8.7 Hz). ^13^C NMR (150 MHz, CDCl_3_) δ: 23.35, 29.77, 43.12, 43.72, 52.95, 57.60, 59.35, 112.03 (^3^*J* = 3.4 Hz), 117.40, 119.04 (^3^*J* = 4.0 Hz), 123.63 (^1^*J* = 267.4 Hz), 124.58, 125.26, 126.07, 126.23, 126.88, 127.39, 129.41, 129.76 (^2^*J* = 32.0 Hz), 132.04, 142.06, 145.74, 151.55. ^19^F NMR (564 MHz, CDCl_3_) δ: −62.72. HRMS (ESI) calcd for: C_25_H_28_F_3_N_4_OS [M+H]^+^: 489.1936, found: 489.1937.

6-(3-(4-(3-chlorophenyl)piperazin-1-yl)propyl)-8-(trifluoromethyl)-quino[3,2-b]benzo[1,4]-thiazine (**13**):

Yield: 75%. M.p.: Yellow oil. ^1^H NMR (CDCl_3_) δ: 2.16 (m, 2H, CH_2_), 2.71 (m, 6H, 3CH_2_), 3.261–3.31 (m, 4H, 2CH_2_), 4.37 (m, 2H, CH_2_), 6.80–6.84 (m, 2H, 2H-Ph), 6.89 (s, 1H, H-7), 7.14 (s, 1H, 1H-Ph), 7.17–7.20 (m, 3H, H-9, H-10, 1H-Ph), 7.31 (t, 1H, H-2, *J* = 7.2 Hz), 7.54–7.55 (m, 2H, H-1, H-3), 7.64 (s, 1H, H-12), 7.72 (d, 1H, H-4, *J* = 7.8 Hz). ^13^C NMR (150 MHz, CDCl_3_) δ: 29.73, 43.712, 48.48, 53.05, 55.91, 112.00 (^3^*J* = 3.9 Hz), 113.97, 115.91, 117.39, 119.12 (^3^*J* = 3.5 Hz), 119.42, 123.07, 123.85, 124.64 (^1^*J* = 270.4 Hz), 125.28, 126.10, 126.25, 126.94, 127.41, 129.47, 129.99 (^2^*J* = 34.5 Hz), 130.05, 132.11, 134.95, 142.02, 145.73, 151.54. ^19^F NMR (564 MHz, CDCl_3_) δ: −62.64. HRMS (ESI) calcd for: C_29_H_27_ClF_3_N_4_S [M+H]^+^: 555.1597, found: 555.1595.

### 4.2. Biological Assays

#### 4.2.1. Cell Line and Culture

The human cell lines MDA-MB-231 (breast cancer), Mia-Paca-2 (pancreatic cancer) A-549 (lung cancer), and HaCaT (immortalized keratinocytes) were sourced from the American Type Culture Collection (ATCC) in Rockville, MD, USA. Culturing conditions varied: A-549, MDA-MB-231, and Mia-Paca-2, HaCaT cells were cultured in DMEM High Glucose (Biowest SAS, France). The growth medium consisted of 10% fetal bovine serum (FBS, Sigma-Aldrich, St. Louis, MO, USA), 20 mM HEPES (Biowest, Nuaillé, France), and antibiotics (100 U/mL of penicillin and 100 μg/mL of streptomycin) from Gibco, Grand Island, NY, USA. All cells were maintained in a humidified incubator at 37 °C with a 5% CO_2_ atmosphere until they reached 80–90% confluence.

#### 4.2.2. MTT Assay

Cytotoxic activity was evaluated using the MTT assay as previously described [69]. The tested compounds were dissolved in DMSO to prepare 10 mM stock solutions and subsequently diluted in culture medium to obtain the required working concentrations. Untreated control cells were incubated in culture medium without test compounds. The final DMSO concentration in the wells was kept constant across all experimental conditions and did not exceed 1% at the highest tested compound concentration. At this DMSO level, no effect on cell viability was observed. Cancer cell lines (MDA-MB-231, Mia-PaCa-2, A-549) and normal HaCaT keratinocytes were seeded in 96-well plates at a density of 1 × 10^4^ cells per well and allowed to adhere overnight. The tested compounds were added at concentrations ranging from 5 to 140 µM and incubated for 72 h. Following incubation, MTT reagent was added and the formation of formazan crystals was quantified spectrophotometrically. Cell viability was expressed as a percentage relative to untreated control cells. All experiments were performed in three independent experiments, each conducted in triplicate (n = 3). IC_50_ values were calculated by nonlinear regression analysis of dose–response curves. Data are presented as mean ± standard deviation (SD). Statistical significance testing was not performed for IC_50_ comparisons, as the analysis was intended to be descriptive and to assess the relative activity of the tested compounds.

#### 4.2.3. In Vitro Antibacterial Studies

##### Bacterial Strains

The antibacterial activity of newly synthesized compounds **3**, **5**, and **8** was evaluated against a panel of reference strains obtained from the American Type Culture Collection (ATCC) and the National Collection of Type Cultures (NCTC), as well as two clinical isolates of *Staphylococcus aureus*. The reference strains included *Staphylococcus aureus* ATCC 6538 and NCTC 4163, *Enterococcus faecalis* ATCC 29212, *Escherichia coli* ATCC 25922, and *Pseudomonas aeruginosa* ATCC 15442. Clinical isolates comprised *S. aureus* 2273 (MSSA) and *S. aureus* T5591 (MRSA, iMLSB phenotype). All strains were stored at −80 °C in appropriate cryoprotective media. Prior to susceptibility testing, bacteria were subcultured twice on Columbia agar (bioMérieux, Marcy-l’Étoile, France) to ensure viability and phenotypic stability.

##### Inoculum Preparation

Fresh bacterial colonies were suspended in sterile 0.85% NaCl and adjusted to a turbidity equivalent to 0.5 McFarland standard (approximately 1 × 10^8^ CFU/mL) using a densitometer. The suspension was subsequently diluted in Mueller–Hinton broth (MHB; bioMérieux, Marcy-l’Étoile, France) to obtain a final inoculum of 5 × 10^5^ CFU/mL, in accordance with standard susceptibility testing procedures.

##### Test Compounds

Tested compounds were dissolved in analytical-grade dimethyl sulfoxide (DMSO; Sigma-Aldrich, St. Louis, MO, USA) and further diluted in MHB. The final concentration of DMSO in test wells did not exceed 5% (*v*/*v*). Two-fold serial dilutions were prepared to achieve final compound concentrations ranging from 1.95 to 250 μg/mL.

MICs were determined by broth microdilution in 96-well plates (Wuxi NEST Biotechnology, Wuxi, China) according to EUCAST recommendations [70] and EN ISO 20776-1:2020 [71]. MIC was defined as the lowest concentration completely inhibiting visible growth (turbidity or a pellet). Each test included a growth control (no compound), sterility control (no inoculum), and a solvent control (DMSO at the corresponding final concentration). DMSO control showed no inhibition vs. growth control.

MBC was defined as the lowest concentration resulting in ≥99.9% (3 log_10_) reduction in viable count relative to the initial inoculum. MBCs were determined by subculturing aliquots from wells without visible growth onto agar plates and recording colony formation after incubation. Experiments were performed in triplicate.

The MBC/MIC ratio was used to classify antimicrobial action. A ratio ≤ 4 was interpreted as bactericidal activity, while >4 indicated a bacteriostatic effect [72].

##### Bactericidal Kinetics Assay

The time-dependent antibacterial activity of compound 3 was assessed using a turbidity-based growth kinetics assay. This analysis was performed exclusively for staphylococcal strains (both reference and clinical isolates), as compound **3** demonstrated the highest activity against this genus.

Kinetic analysis was not performed for enterococci; therefore, conclusions regarding *E. faecalis* remain preliminary due to the inclusion of a single strain only.

Wells containing the bacterial inoculum without compound **3** served as positive growth controls, whereas wells containing MHB supplemented with compound 3 but lacking bacterial inoculation were used for blank correction. Optical density at 595 nm (OD_595_) was measured at 37 °C every 2 h for 18 h using a microplate reader (Thermo Scientific, Waltham, MA, USA).

#### 4.2.4. Statistical Analysis

Statistical analysis was performed using GraphPad Prism 8.0.1 software. Data are presented as mean ± SD from at least three independent experiments. Differences between groups were analyzed using one-way analysis of variance (ANOVA) followed by Dunnett’s post hoc test. The significance level, i.e., *p* value < 0.05 was considered statistically significant.

## 5. Conclusions

In the present study, an efficient method for the synthesis of 6*H*-8-trifluoromethylquinobenzothiazine was developed and successfully used to obtain a series of eleven new N-substituted derivatives structurally related to trifluoromethyl-substituted phenothiazine drugs. Comprehensive spectroscopic characterization confirmed the proposed structures and substitution patterns of the synthesized compounds. Biological evaluation revealed that selected derivatives exhibit biologically relevant activities in both, anticancer and antibacterial models. Compounds **8** and **12** demonstrated the most balanced anticancer profiles, combining low micromolar cytotoxicity against lung and pancreatic cancer cell lines with moderate selectivity toward malignant cells relative to normal keratinocytes. Although compound **6** displayed lower intrinsic cytotoxic potency, its lack of toxicity toward normal cells resulted in a favorable selectivity index, highlighting this scaffold as a promising starting point for further optimization aimed at improving efficacy while preserving safety. In parallel, antibacterial screening identified compound **3** as a potent agent against Gram-positive bacteria, including methicillin-resistant Staphylococcus aureus. Its low MIC values, bactericidal MBC/MIC ratios, and concentration-dependent inhibition of bacterial growth kinetics underscore the relevance of the quinobenzothiazine framework for antimicrobial drug discovery, particularly in the context of increasing resistance among staphylococcal pathogens. Overall, the presented results demonstrate that structural modification of trifluoromethyl-substituted phenothiazines by incorporation of a quinoline moiety yields compounds with diversified and therapeutically relevant biological activities. Future studies will focus on expanding the library of 8-trifluoromethylquinobenzothiazine derivatives to include analogues bearing piperazine and piperidine ring substituents, elucidating their anticancer mechanism of action, and determining lipophilicity parameters to support further structure–activity relationship optimization.

## Figures and Tables

**Table 1:** Antiproliferative activity against human cancer cell lines and cytotoxicity toward normal HaCaT keratinocytes of synthesized compounds **1**–**13** and reference drugs by the MTT assay ^a^.

Compound	Cancer Cells						Normal Cells
	MDA-MB-231 ^b^		Mia-PaCa-2 ^c^		A-549 ^d^		HaCaT ^e^
	IC_50_^f^	SI ^g^	IC_50_	SI	IC_50_	SI	IC_50_
**1**	>100	<0.47	>100	<0.47	>100	<0.47	46.88 ± 4.69
**2**	>100	1	>100	1	>100	1	>100
**3**	78.75 ± 5.45	0.03	5.32 ± 0.38	0.46	4.90 ± 1.48	0.51	2.48 ± 0.03
**4**	>100	<0.55	>100	<0.55	>100	<0.55	55.44 ± 6.58
**5**	24.42 ± 3.40	1.04	19.56 ± 2.25	1.30	24.13 ± 5.45	1.06	25.51 ± 2.86
**6**	>100	1	43.38 ± 3.85	>2.31	47.75 ± 2.28	>2.09	>100
**7**	45.14 ± 4.32	0.46	31.28 ± 2.47	0.66	49.90 ± 4.11	0.41	20.59 ± 2.28
**8**	9.96 ± 0.05	1.31	6.98 ± 2.13	1.87	7.52 ± 0.59	1.74	13.06 ± 2.51
**9**	>100	<0.12	>100	<0.12	>100	<0.12	12.22 ± 1.00
**10**	>100	<0.38	>100	<0.38	>100	<0.38	37.77 ± 6.51
**11**	17.88 ± 4.67	0.63	12.18 ± 4.92	0.93	11.31 ± 3.93	0.99	11.29 ± 2.65
**12**	10.76 ± 0.42	0.67	5.16 ± 0.53	1.40	4.22 ± 0.10	1.71	7.21 ± 3.61
**13**	>100	1	>100	1	>100	1	>100
Doxorubicin ^h^	0.5 ± 0.13	0.60	24.2 ± 0.5	0.01	0.26 ± 0.09	1.15	0.3 ± 0.11
Cisplatin ^i^	1.9 ± 0.42	3.31	3.15 ± 0.09	2	3.17 ± 1.24	1.98	6.3 ± 0.70

^a^ Data are expressed as mean values ± SD (standard deviation), ^b^ Human breast adenocarcinoma (MDA-MB-231), ^c^ Human pancreatic cancer (Mia-PaCa-2), ^d^ Human lung carcinoma (A-549), ^e^ Human immortal keratinocyte cell line from adult human skin (HaCaT). ^f^ IC_50_ (µM)—the half-maximal growth inhibitory concentration after cultured the cells for 72 h with the individual compound. ^g^ The SI (Selectivity Index) was calculated using formula: SI = IC_50_ for normal cell line/IC_50_ cancer cell line. ^h, i^ The reference compounds.

**Table 2:** Antibacterial activity of 8-trifluoromethylquinobenzothiazines **3**, **5**, and **8** against reference strains and clinical isolates.

	Substance	3			5			8			CIP
		MIC	MBC	MBC/MIC	MIC	MBC	MBC/MIC	MIC	MBC	MBC/MIC	MIC
Bacteria		µg/mL			µg/mL			µg/mL			µg/mL
*S. aureus* ATCC6538		7.8	15.6	2	250	250	1	125	125	1	0.125
*S. aureus* NCTC 4163		15.6	15.6	1	250	250	1	250	250	1	0.25
*S. aureus* 2273 MSSA		15.6	15.6	1	250	250	1	125	250	2	0.5
*S. aureus* T5591 MRSA		15.6	15.6	1	>250	-	-	250	250	1	0.5
*E. faecalis* ATCC 29212		15.6	15.6	1	>250	-	-	125	125	1	0.5
*E. coli* ATCC 25922		125	125	1	>250	-	-	>250	-	-	0.015
*P. aeruginosa* ATCC 15442		>250	-	-	>250	-	-	>250	-	-	0.06

Minimum inhibitory concentrations (MIC), minimum bactericidal concentrations (MBC) and MBC/MIC ratios were determined by microdilution method in Mueller–Hinton broth. The tested concentration range was 0.97–250 µg/mL. Values reported as “>250” indicate no inhibition at the highest concentration tested; in these cases, MBC was not determined (−). CIP; ciprofloxacin, MSSA; methicillin-sensitive *S. aureus*, MRSA; methicillin-resistant *S. aureus*, it means resistance to most be-ta-lactam antibiotics, including penicillins, carbapenems, monobactams and cephalosporins (except fifth-generation cephalo-sporins), due to the production of a modified penicillin-binding protein (PBP2a/PBP2’), which is encoded by the mecA or mecC gene.

## Data Availability

The original contributions presented in this study are included in the article. Further inquiries can be directed to the corresponding author.

## Supplementary Materials

The following supporting information can be downloaded at: https://www.mdpi.com/article/10.3390/ph19030422/s1, Table S1: The proton–proton correlation of compound **2**; Table S2: The proton–carbon correlation of compound **2**. ^1^H NMR and ^13^C NMR spectra and HR MS of compounds **1**–**13**.

## References

[1] Bray F., Laversanne M., Sung H., Ferlay J., Siegel R.L., Soerjomataram I., Jemal A. (2024). Global Cancer Statistics 2022: GLOBOCAN Estimates of Incidence and Mortality Worldwide for 36 Cancers in 185 Countries. CA. Cancer J. Clin..

[2] Belay W.Y., Getachew M., Tegegne B.A., Teffera Z.H., Dagne A., Zeleke T.K., Wondm S.A., Abebe R.B., Gedif A.A., Fenta A. (2025). Antimicrobial Resistance with a Focus on Antibacterial, Antifungal, Antimalarial, and Antiviral Drugs Resistance, Its Threat, Global Priority Pathogens, Prevention, and Control Strategies: A Review. Ther. Adv. Infect. Dis..

[3] Ohlow M.J., Moosmann B. (2011). Phenothiazine: The Seven Lives of Pharmacology’s First Lead Structure. Drug Discov. Today.

[4] Mosnaim A.D., Ranade V.V., Wolf M.E., Puente J., Antonieta Valenzuela M. (2006). Phenothiazine Molecule Provides the Basic Chemical Structure for Various Classes of Pharmacotherapeutic Agents. Am. J. Ther..

[5] Shen W.W. (1999). A History of Antipsychotic Drug Development. Compr. Psychiatry.

[6] Ban T.A. (2007). Fifty Years Chlorpromazine: A Historical Perspective. Neuropsychiatr. Dis. Treat..

[7] Posso M.C., Domingues F.C., Ferreira S., Silvestre S. (2022). Development of Phenothiazine Hybrids with Potential Medicinal Interest: A Review. Molecules.

[8] Jana S., Empel C., Pei C., Vinh Nguyen T., Koenigs R.M. (2020). Gold-Catalyzed C−H Functionalization of Phenothiazines with Aryldiazoacetates. Adv. Synth. Catal..

[9] Zubaș A., Ghinet A., Farce A., Dubois J., Bîcu E. (2023). Phenothiazine- and Carbazole-Cyanochalcones as Dual Inhibitors of Tubulin Polymerization and Human Farnesyltransferase. Pharmaceuticals.

[10] Zhang Z., Zhou L., Xie N., Nice E.C., Zhang T., Cui Y., Huang C. (2020). Overcoming Cancer Therapeutic Bottleneck by Drug Repurposing. Signal Transduct. Target. Ther..

[11] Xia Y., Sun M., Huang H., Jin W.-L. (2024). Drug Repurposing for Cancer Therapy. Signal Transduct. Target. Ther..

[12] Natte K., Jagadeesh R.V., He L., Rabeah J., Chen J., Taeschler C., Ellinger S., Zaragoza F., Neumann H., Brückner A. (2016). Palladium-Catalyzed Trifluoromethylation of (Hetero)Arenes with CF3Br. Angew. Chem. Int. Ed..

[13] Levin M.D., Chen T.Q., Neubig M.E., Hong C.M., Theulier C.A., Kobylianskii I.J., Janabi M., O’Neil J.P., Toste F.D. (2017). A Catalytic Fluoride-Rebound Mechanism for C(Sp3)-CF3 Bond Formation. Science.

[14] Poła A., Palko-Łabuz A., Środa-Pomianek K. (2021). Theoretical Study of 2-(Trifluoromethyl)Phenothiazine Derivatives with Two Hydroxyl Groups in the Side Chain-DFT and QTAIM Computations. Molecules.

[15] Johnson J.W., Cain K.W., Dunlap T.B., Naumiec G.R. (2018). Improved Synthesis of 2-Trifluoromethyl-10-Aminopropylphenothiazine: Making 2-Trifluoromethyl-10-Aminopropylphenothiazine Readily Available for Calmodulin Purification. ACS Omega.

[16] Wu C.-H., Bai L.-Y., Tsai M.-H., Chu P.-C., Chiu C.-F., Chen M.Y., Chiu S.-J., Chiang J.-H., Weng J.-R. (2016). Pharmacological Exploitation of the Phenothiazine Antipsychotics to Develop Novel Antitumor Agents–A Drug Repurposing Strategy. Sci. Rep..

[17] Schipper L.J., Zeverijn L.J., Garnett M.J., Voest E.E. (2022). Can Drug Repurposing Accelerate Precision Oncology?. Cancer Discov..

[18] Vesenbeckh S., Krieger D., Bettermann G., Schönfeld N., Rüssmann H., Mauch H., Bauer T. (2015). Neuroleptic Drugs as Potential Adjuvants in the Treatment of MDR-TB: Minimal Inhibitory Concentrations (MICs) of Different Phenothiazines against M. Tuberculosis. Eur. Respir. J..

[19] Dinić J., Efferth T., García-Sosa A.T., Grahovac J., Padrón J.M., Pajeva I., Rizzolio F., Saponara S., Spengler G., Tsakovska I. (2020). Repurposing Old Drugs to Fight Multidrug Resistant Cancers. Drug Resist. Updates.

[20] Macdonald R., Watts T.P.S. (1959). Trifluoperazine Dihydrochloride (“Stelazine”) in Paranoid Schizophrenia. Br. Med. J..

[21] Davies J.I., Hansen J.M., Angell S.N. (1959). Triflupromazine (Vesprin^®^) in Anaesthesia: A Clinical Evaluation. Can. Anaesth. Soc. J..

[22] López-Muñoz F., Álamo C. (2011). Neurobiological Background for the Development of New Drugs in Schizophrenia. Clin. Neuropharmacol..

[23] Varga B., Csonka Á., Csonka A., Molnár J., Amaral L., Spengler G. (2017). Possible Biological and Clinical Applications of Phenothiazines. Anticancer Res..

[24] Dastidar S.G., Debnath S., Mazumdar K., Ganguly K., Chakrabarty A.N. (2004). Triflupromazine: A Microbicide Non-Antibiotic Compound. Acta Microbiol. Immunol. Hung..

[25] Wang Q., Zhang Y., Zhang Y., Zhai Y., Zhou X., Qin Y., Ding M., Tian Y., Zhang Z., Zhang P. (2025). Triflupromazine and Tranylcypromine Alleviate Primary Cisplatin Resistance in Lung Adenocarcinoma by Promoting LDHA-Mediated AMBRA1 Ubiquitination. Biochem. Pharmacol..

[26] Sultan N.S., Shoukry A.A., Rashidi F.B., Elhakim H.K.A. (2024). Biological Applications, In Vitro Cytotoxicity, Cellular Uptake, and Apoptotic Pathway Studies Induced by Ternary Cu (II) Complexes Involving Triflupromazine with Biorelevant Ligands. Cell Biochem. Biophys..

[27] Yeh C., Lee W., Yu T., Hsu Y., Lan M., Lan M. (2023). Antipsychotic Drug Trifluoperazine as a Potential Therapeutic Agent against Nasopharyngeal Carcinoma. Head Neck.

[28] Huang C., Lan W., Fraunhoffer N., Meilerman A., Iovanna J., Santofimia-Castaño P. (2019). Dissecting the Anticancer Mechanism of Trifluoperazine on Pancreatic Ductal Adenocarcinoma. Cancers.

[29] Xia Y., Jia C., Xue Q., Jiang J., Xie Y., Wang R., Ran Z., Xu F., Zhang Y., Ye T. (2019). Antipsychotic Drug Trifluoperazine Suppresses Colorectal Cancer by Inducing G0/G1 Arrest and Apoptosis. Front. Pharmacol..

[30] Pinheiro T., Otrocka M., Seashore-Ludlow B., Rraklli V., Holmberg J., Forsberg-Nilsson K., Simon A., Kirkham M. (2017). A Chemical Screen Identifies Trifluoperazine as an Inhibitor of Glioblastoma Growth. Biochem. Biophys. Res. Commun..

[31] Xia Y., Xu F., Xiong M., Yang H., Lin W., Xie Y., Xi H., Xue Q., Ye T., Yu L. (2021). Repurposing of Antipsychotic Trifluoperazine for Treating Brain Metastasis, Lung Metastasis and Bone Metastasis of Melanoma by Disrupting Autophagy Flux. Pharmacol. Res..

[32] Kang S., Hong J., Lee J.M., Moon H.E., Jeon B., Choi J., Yoon N.A., Paek S.H., Roh E.J., Lee C.J. (2017). Trifluoperazine, a Well-Known Antipsychotic, Inhibits Glioblastoma Invasion by Binding to Calmodulin and Disinhibiting Calcium Release Channel IP3R. Mol. Cancer Ther..

[33] Park J., Jeong J.Y., Kang S.S. (2022). COX-2 Acts as a Key Mediator of Trifluoperazine-Induced Cell Death in U87MG Glioma Cells. Anticancer Res..

[34] Jeong J.Y., Park H., Yoo H., Kim E.-J., Jeon B., Lee J.D., Kang D., Lee C.J., Paek S.H., Roh E.J. (2022). Trifluoperazine and Its Analog Suppressed the Tumorigenicity of Non-Small Cell Lung Cancer Cell; Applicability of Antipsychotic Drugs to Lung Cancer Treatment. Biomedicines.

[35] Kiani A., Nik S.H., Khodadoost A., Salimi A., Pourahmad J. (2020). Trifluoperazine an Antipsychotic Drug and Inhibitor of Mitochondrial Permeability Transition Protects Cytarabine and Ifosfamide-Induced Neurotoxicity. Drug Res..

[36] Budd G.T., Bukowski R.M., Lichtin A., Bauer L., Van Kirk P., Ganapathi R. (1993). Phase II Trial of Doxorubicin and Trifluoperazine in Metastatic Breast Cancer. Investig. New Drugs.

[37] Piccini L.E., Castilla V., Damonte E.B. (2022). Inhibition of Dengue Virus Infection by Trifluoperazine. Arch. Virol..

[38] Duarte D., Vale N. (2022). Antipsychotic Drug Fluphenazine against Human Cancer Cells. Biomolecules.

[39] Miron-Ocampo A., Beattie S.R., Guin S., Conway T., Meyers M.J., Moye-Rowley W.S., Krysan D.J. (2023). CWHM-974 Is a Fluphenazine Derivative with Improved Antifungal Activity against Candida Albicans Due to Reduced Susceptibility to Multidrug Transporter-Mediated Resistance Mechanisms. Antimicrob. Agents Chemother..

[40] Xu F., Xia Y., Feng Z., Lin W., Xue Q., Jiang J., Yu X., Peng C., Yang Y., Wei Y. (2019). Repositioning Antipsychotic Fluphenazine Hydrochloride for Treating Triple Negative Breast Cancer with Brain Metastases and Lung Metastases. Am. J. Cancer Res..

[41] Ochu S.R., Edache E.I., Shafiu S., Idowu A.E. (2017). Docking Studies of a Series of Fluphenazine as Potential 1RE1(X-Ray Crystal Structure of Caspase-3) Inhibitors: A Rational Approach to Anticancer Drug Design. J. Adv. Chem. Sci..

[42] Babalola B.A., Malik M., Sharma L., Olowokere O., Folajimi O. (2024). Exploring the Therapeutic Potential of Phenothiazine Derivatives in Medicinal Chemistry. Results Chem..

[43] Zhang X., Shi X., Zhang X., Zhang Y., Yu S., Zhang Y., Liu Y. (2024). Repositioning Fluphenazine as a Cuproptosis-Dependent Anti-Breast Cancer Drug Candidate Based on TCGA Database. Biomed. Pharmacother..

[44] Su C., Cheng C., Rong Z., Yang J., Li Z., Yao J., Liu A., Yang L., Zhao M. (2023). Repurposing Fluphenazine as an Autophagy Modulator for Treating Liver Cancer. Heliyon.

[45] Cheon J.H., Lee B.M., Kim H.S., Yoon S. (2016). Highly Halaven-Resistant KBV20C Cancer Cells Can Be Sensitized by Co-Treatment with Fluphenazine. Anticancer Res..

[46] Dastidar S.G., Chaudhury A., Annadurai S., Roy S., Mookerjee M., Chakrabarty A.N. (1995). In Vitro and in Vivo Antimicrobial Action of Fluphenazine. J. Chemother. Florence Italy.

[47] Bourlioux P., Moreaux J.M., Su W.J., Boureau H. (1992). In Vitro Antimicrobial Activity of 18 Phenothiazine Derivatives: Structure-Activity Relationship. APMIS. Suppl..

[48] Jeleń M., Morak-Młodawska B., Korlacki R. (2023). Anticancer Activities of Tetra-, Penta-, and Hexacyclic Phenothiazines Modified with Quinoline Moiety. J. Mol. Struct..

[49] Jeleń M., Pluta K., Latocha M., Morak-Młodawska B., Suwińska K., Kuśmierz D. (2019). Evaluation of Angularly Condensed Diquinothiazines as Potential Anticancer Agents. Bioorg. Chem..

[50] Jeleń M., Pluta K., Zimecki M., Morak-Młodawska B., Artym J., Kocięba M., Kochanowska I. (2016). Synthesis and Biological Evaluation of Novel Propargylquinobenzothiazines and Their Derivatives as Potential Antiproliferative, Anti-Inflammatory, and Anticancer Agents. J. Enzym. Inhib. Med. Chem..

[51] Jeleń M., Pluta K., Szmielew M., Morak-Młodawska B., Herman K., Giercuszkiewicz K., Kasprzycka A., Skonieczna M. (2023). 14-Substituted Diquinothiazines as a New Group of Anticancer Agents. Molecules.

[52] Jeleń M., Pluta K., Zimecki M., Morak-Młodawska B., Artym J., Kocięba M. (2013). Synthesis and Selected Immunological Properties of Substituted Quino [3,2-b]Benzo[1,4]Thiazines. Eur. J. Med. Chem..

[53] Jeleń M., Otto-Ślusarczyk D., Morak-Młodawska B., Struga M. (2024). Novel Tetracyclic Azaphenothiazines with the Quinoline Ring as New Anticancer and Antibacterial Derivatives of Chlorpromazine. Int. J. Mol. Sci..

[54] Lodarski K., Jończyk J., Guzior N., Bajda M., Gładysz J., Walczyk J., Jeleń M., Morak-Młodawska B., Pluta K., Malawska B. (2015). Discovery of Butyrylcholinesterase Inhibitors among Derivatives of Azaphenothiazines. J. Enzym. Inhib. Med. Chem..

[55] Artym J., Kochanowska I.E., Kocięba M., Zaczyńska E., Zimecki M., Jeleń M., Morak-Młodawska B., Pluta K. (2016). Selected Azaphenothiazines Inhibit Delayed Type Hypersensitivity and Carrageenan Reaction in Mice. Int. Immunopharmacol..

[56] Artym J., Kocięba M., Zaczyńska E., Zimecki M., Jeleń M., Morak-Młodawska B., Pluta K., Kaleta-Kuratewicz K., Madej J.P., Kuropka P. (2018). Topically Applied Azaphenothiazines Inhibit Contact Sensitivity to Oxazolone in Mice. Histol. Histopathol..

[57] Sylvester P.W., Satyanarayanajois S.D. (2011). Optimization of the Tetrazolium Dye (MTT) Colorimetric Assay for Cellular Growth and Viability. Drug Design and Discovery: Methods and Protocols.

[58] Mosmann T. (1983). Rapid Colorimetric Assay for Cellular Growth and Survival: Application to Proliferation and Cytotoxicity Assays. J. Immunol. Methods.

[59] Agarwal N.L., Sharma A.K., Jamwal R.S., Atal C.K., Torres T. (1987). A Convenient and Single-Step Synthesis of Substituted 12H-Quinoxalino-[2,3-b][1,4]Benzothiazines and -Benzoxazines. Liebigs Ann. Chem..

[60] Pluta K., Morak-Młodawska B., Jeleń M. (2020). The Smiles Rearrangement in the Syntheses of Azaphenothiazines. Part II. The Review of the Various Types of Phenyl Azinyl and Diazinyl Sulfides Undergoing This Rearrangement. J. Mol. Struct..

[61] Pluta K., Jeleń M. (2009). Synthesis of Quinobenzo-1,4-Thiazines from Diquino-1,4-Dithiin and 2,2′-Dichloro-3,3′-Diquinolinyl Disulfide. Heterocycles.

[62] Pluta K., Jeleń M., Suwińska K., Besnard C., Morak-Młodawska B. (2012). The Structures of 8- and 10-Trifluoromethylquino[3,2-b]Benzo[1,4]Thiazines and Their Benzyl Derivatives. HETEROCYCLES.

[63] Dai X.-J., Zhao L.-J., Yang L.-H., Guo T., Xue L.-P., Ren H.-M., Yin Z.-L., Xiong X.-P., Zhou Y., Ji S.-K. (2023). Phenothiazine-Based LSD1 Inhibitor Promotes T-Cell Killing Response of Gastric Cancer Cells. J. Med. Chem..

[64] Strobl J.S., Peterson V.A. (1992). Tamoxifen-Resistant Human Breast Cancer Cell Growth: Inhibition by Thioridazine, Pimozide and the Calmodulin Antagonist, W-13. J. Pharmacol. Exp. Ther..

[65] Shen J., Ma B., Zhang X., Sun X., Han J., Wang Y., Chu L., Xu H., Yang Y. (2017). Thioridazine Has Potent Antitumor Effects on Lung Cancer Stem-like Cells. Oncol. Lett..

[66] Qian G., Dai L., Yu T. (2019). Thioridazine Sensitizes Cisplatin Against Chemoresistant Human Lung and Ovary Cancer Cells. DNA Cell Biol..

[67] Goyette M.-A., Cusseddu R., Elkholi I., Abu-Thuraia A., El-Hachem N., Haibe-Kains B., Gratton J.-P., Côté J.-F. (2019). AXL Knockdown Gene Signature Reveals a Drug Repurposing Opportunity for a Class of Antipsychotics to Reduce Growth and Metastasis of Triple Negative Breast Cancer. Oncotarget.

[68] Asantewaa A.A., Yartey S.N.-A., Donkor E.S. (2025). A Systematic Review and Meta-Analysis of the Risk of Mortality Associated with Methicillin-Resistant *Staphylococcus aureus* Clones. J. Glob. Antimicrob. Resist..

[69] Otto-Ślusarczyk D., Mielczarek-Puta M., Graboń W. (2021). The Real Cytotoxic Effect of Artemisinins on Colon Cancer Cells in a Physiological Cell Culture Setting. How Composition of the Culture Medium Biases Experimental Findings. Pharmaceuticals.

[70] MIC Determination. https://www.eucast.org/bacteria/methodology-and-instructions/mic-determination/.

[71] (2019). Susceptibility testing of infectious agents and evaluation of performance of antimicrobial susceptibility test devices—Part 1: Broth micro-dilution reference method for testing the in vitro activity of antimicrobial agents against rapidly growing aerobic bacteria involved in infectious diseases. https://www.iso.org/standard/70464.html.

[72] Pankey G.A., Sabath L.D. (2004). Clinical Relevance of Bacteriostatic versus Bactericidal Mechanisms of Action in the Treatment of Gram-Positive Bacterial Infections. Clin. Infect. Dis..

