# Supplementary material for: Novel 8-trifluoromethylquinobenzothiazines—Synthesis and Evaluation for Antiproliferative and Antibacterial Activity
Source: Pharmaceuticals (Basel). 2026 Mar 4;19(3):422. doi: 10.3390/ph19030422 (PMC13029083; doi:10.3390/ph19030422)
Supplement: Supplementary file 1 [file pharmaceuticals-19-00422-s001.zip › pharmaceuticals-4177112-supplementary.pdf]

Novel 8-trifluoromethylquinobenzothiazines - Synthesis and Evaluation for Antiproliferative and Antibacterial Activity

Daria Klimoszek [1], Anna Majewska [2], Małgorzata Jeleń [3,*], Marta Struga [4], Beata Morak-Młodawska[3] and Małgorzata Dołowy [1]

[1] Department of Analytical Chemistry, Faculty of Pharmaceutical Sciences in Sosnowiec, Medical University of Silesia in Katowice, Jagiellońska Street 4, 41-200 Sosnowiec, Poland; d201204@365.sum.edu.pl (D.K.); mdolowy@sum.edu.pl (M.D.)
[2] Chair and Department of Medical Microbiology, Medical University of Warsaw, Chalubinski 5 Str., 02-004 Warsaw, Poland; anna.majewska@wum.edu.pl (A.M.)
[3] Department of Organic Chemistry, Faculty of Pharmaceutical Sciences, The Medical University of Silesia, Jagiellońska 4, 41-200 Sosnowiec, Poland; bmlodawska@sum.edu.pl (B.M.-M.)
[4] Chair and Department of Biochemistry, Medical University of Warsaw, 02-097 Warsaw, Poland; marta.struga@wum.edu.pl (M.S.)

* Correspondence: manowak@sum.edu.pl (M.J.)

Content:

Table S1. The proton–proton correlation of compound **2**.

Table S2. The proton–carbon correlation of compound **2**.

^{1}H NMR and ^{13}C NMR spectra and HR MS of compounds **1 - 13**.

Table S1. The proton–proton correlation of compound **2**.

^{1}H NMR (ppm)	ROESY	COSY
3.65 CH_3	7.10	
7.10 H-7	3.65	7.19-7.23
7.19-7.23 H-9, H-10		7.10
7.32 H-2		7.54-7.57
7.54-7.57 H-1, H-3		7.32
7.69 H-12		
7.81 H-4		7.32, 7.54-7.57

Table S2. The proton–carbon correlation of compound **2**.

^{13}C NMR	HSQC	HMBC
33.87	3.65	
111.81	7.10 C-7	7.19-7.23
117.46		7.19-7.23 C-10a
119.30	7.19-7.23 C-9	
123.99		7.10, 7.19-7.23 C-8
124.65	7.32 C-2	
125.35		7.69, 3.65 C-5a
125.98		7.81 C-4a
126.33	7.54-7.57 C-3	
126.72	7.19-7.23 C-10	
127.48	7.81 C-4	7.54–7.57
129.49	7.54-7.57 C-1	
130.03		
132.40	7.69 C-12	
143.37		7.10 C-6a
145.74		7.54-7.57, 7.69 C-12a
152.48		7.69 C-11a

1

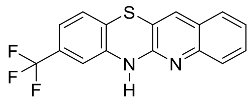

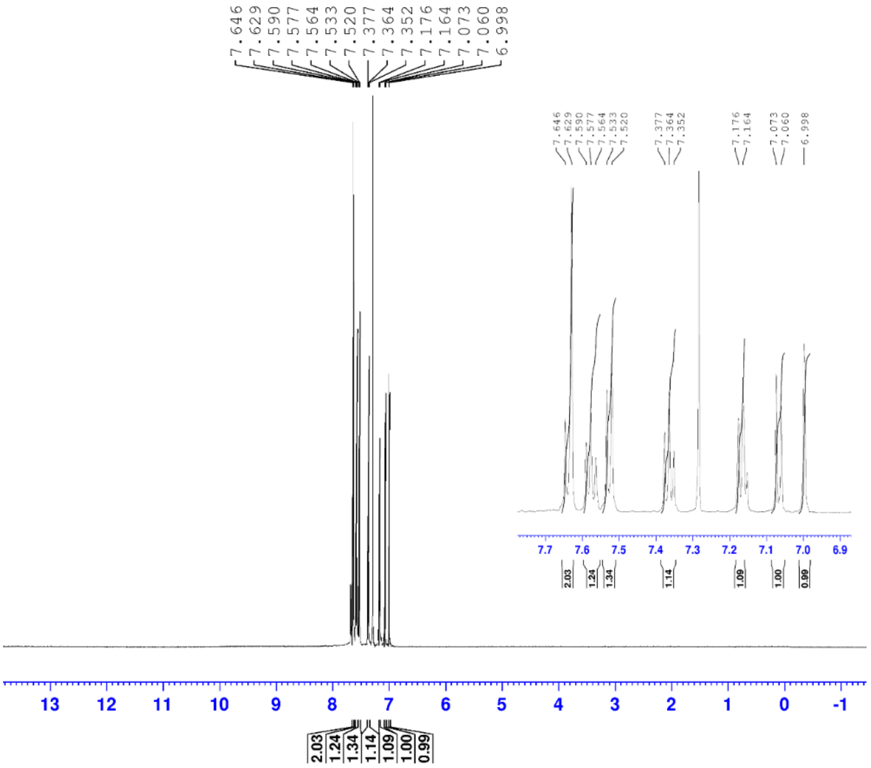

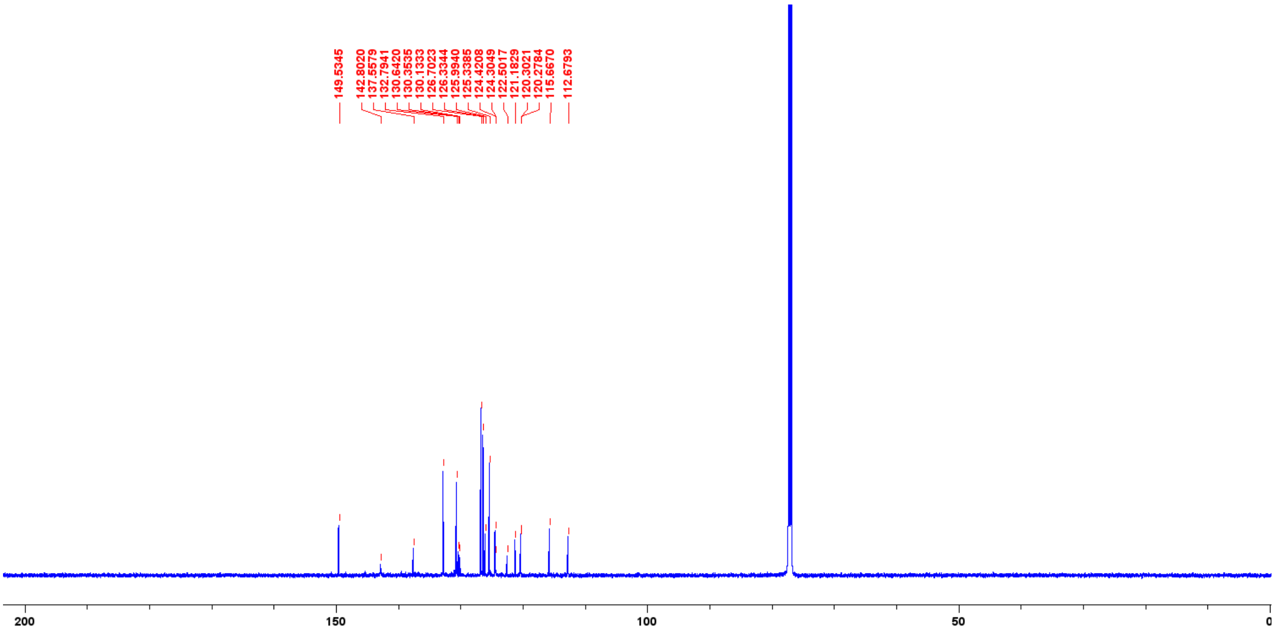

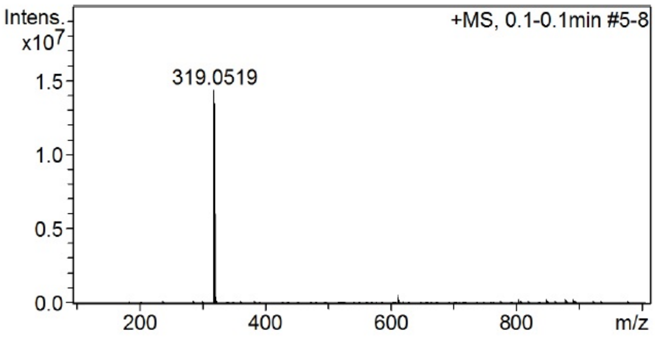

#	m/z	Res.	S/N	I	I %	FWHM
1	319.0519	37286	31884.6	14357121	100.0	0.0086
2	320.0546	30214	5057.8	2285037	15.9	0.0106

2

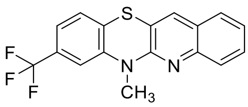

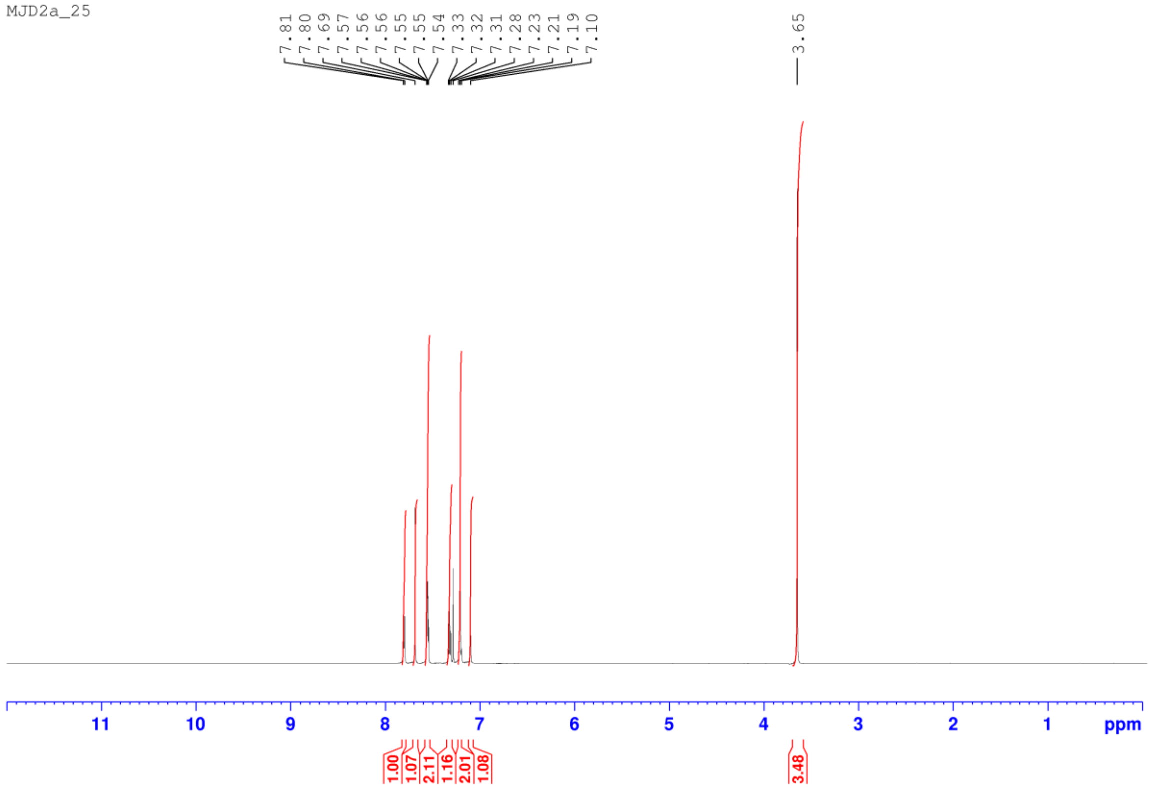

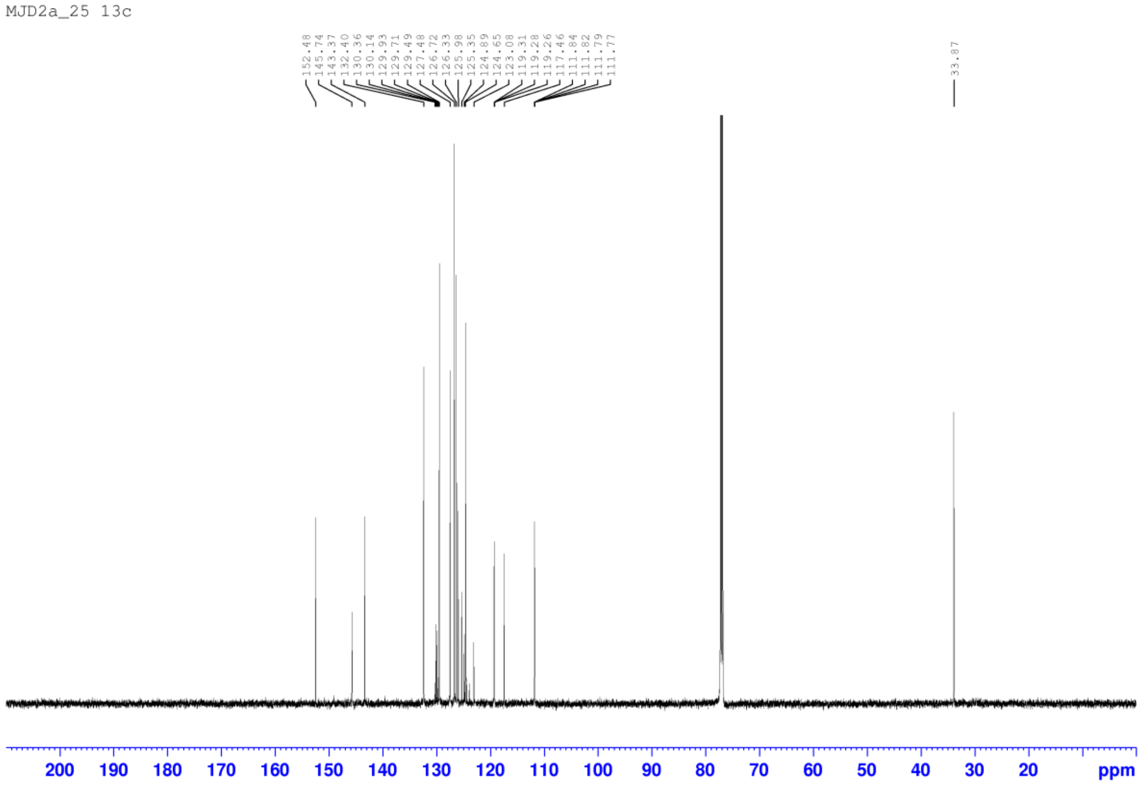

+MS, 0.1-0.2min #5-12

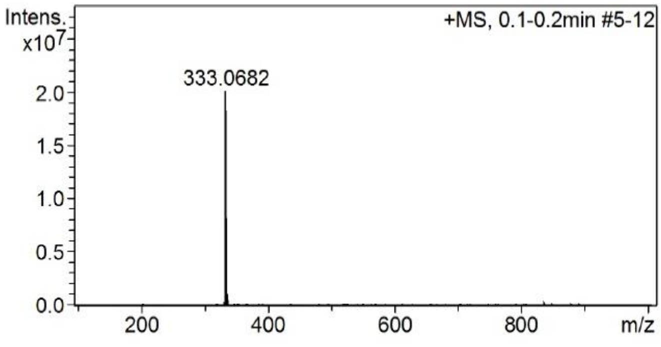

#	m/z	Res.	S/N	I	I %	FWHM
1	333.0682	16210	33576.1	20123098	100.0	0.0205
2	334.0706	35538	9350.6	5601697	27.8	0.0094

3

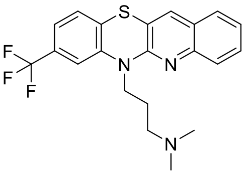

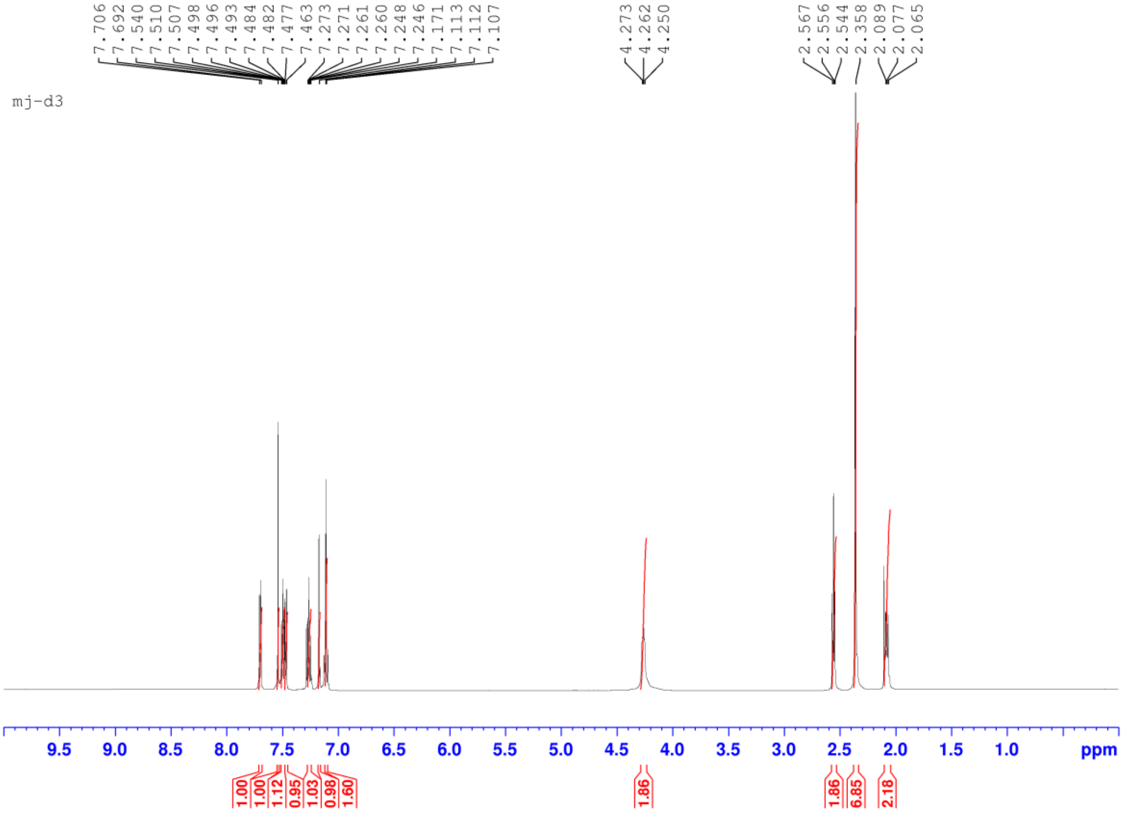
7.706
7.692
7.540
7.510
7.507
7.498
7.496
7.493
7.484
7.482
7.477
7.463
7.273
7.271
7.261
7.260
7.248
7.246
7.171
7.113
7.112
7.107
4.273
4.262
4.250
2.567
2.556
2.544
2.358
2.089
2.077
2.065
mj-d3
9.5
9.0
8.5
8.0
7.5
7.0
6.5
6.0
5.5
5.0
4.5
4.0
3.5
3.0
2.5
2.0
1.5
1.0
ppm
1.00
1.00
1.12
0.95
1.03
0.98
1.60
1.86
1.86
6.85
2.18

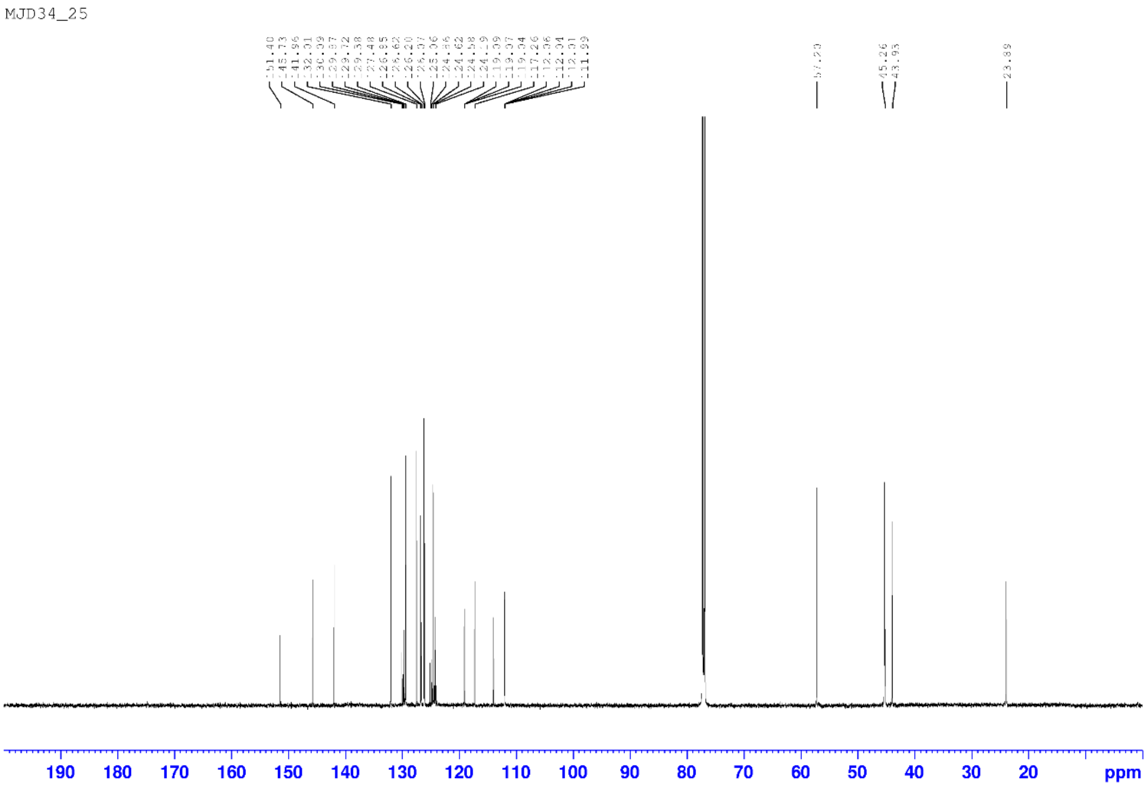

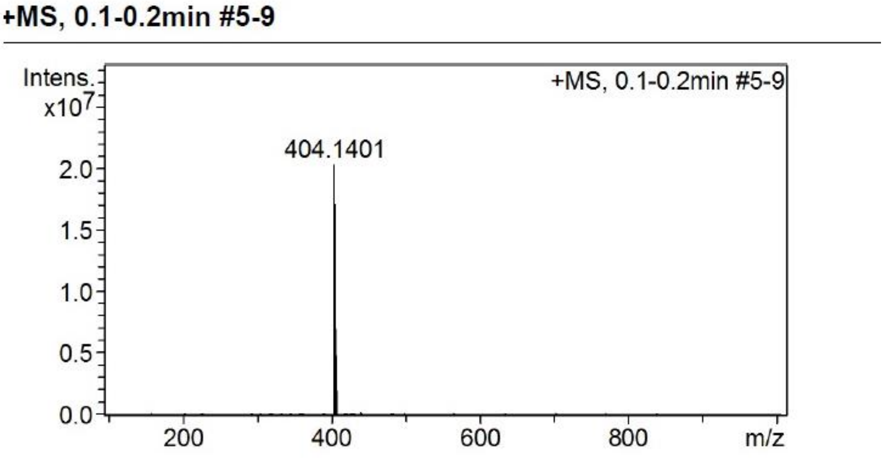

#	m/z	Res.	S/N	I	I %	FWHM
1	404.1401	128529	66670.8	20377596	100.0	0.0031

4

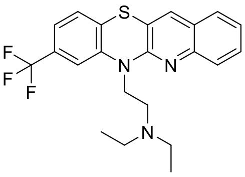

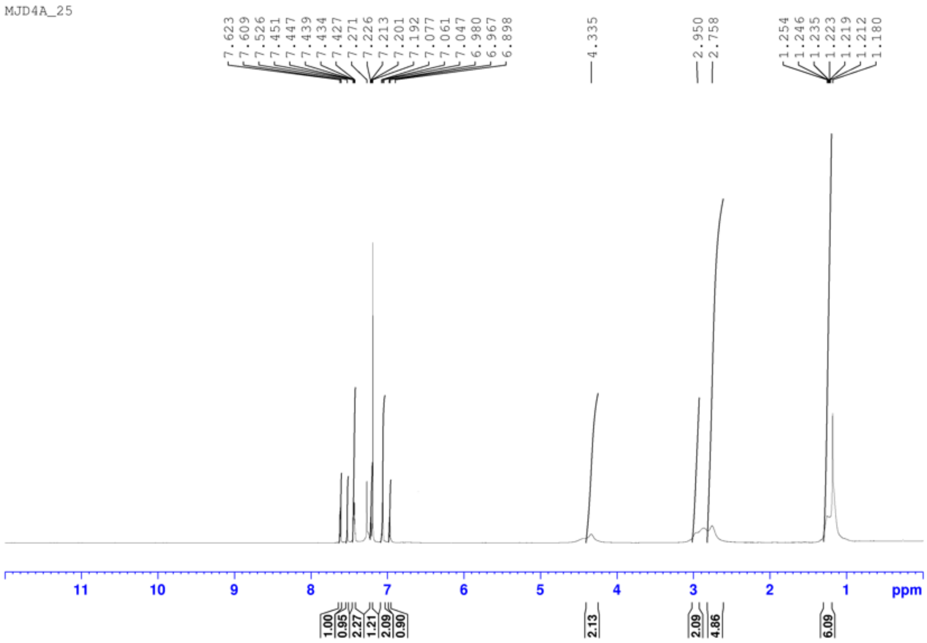
MJD4A_25
7.623
7.609
7.526
7.451
7.447
7.439
7.434
7.427
7.271
7.226
7.213
7.201
7.192
7.077
7.061
7.047
6.980
6.967
6.898
4.335
2.950
2.758
1.254
1.246
1.235
1.223
1.219
1.212
1.180
11
10
9
8
7
6
5
4
3
2
1
ppm
1.00
0.95
2.27
1.21
2.09
0.90
2.13
2.09
4.86
6.09

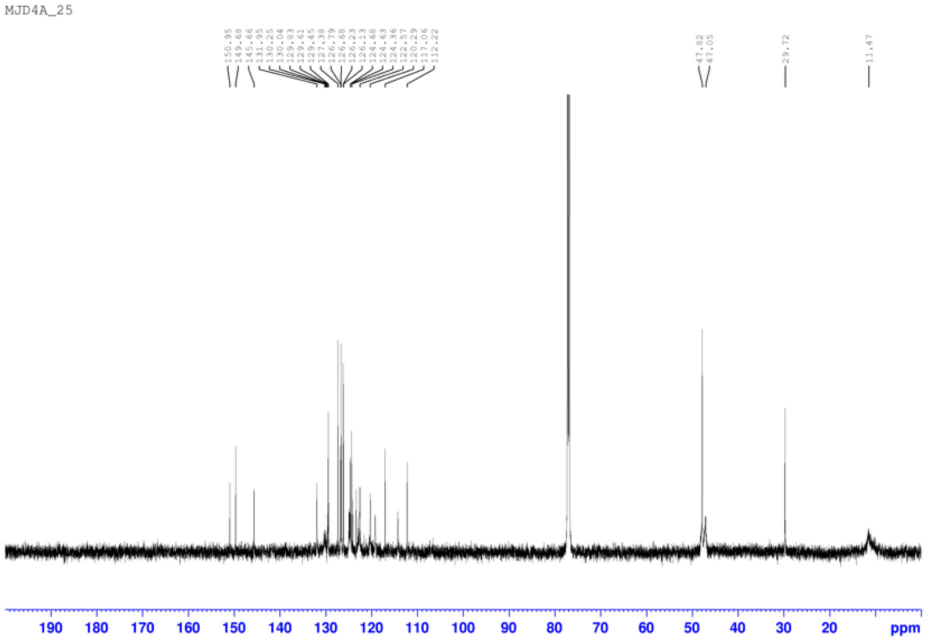
MJD4A_25
150.95
149.68
145.66
131.95
130.25
130.04
129.83
129.61
129.45
127.38
126.79
126.68
126.23
126.13
124.68
124.63
124.36
122.57
120.59
117.06
112.22
47.82
47.05
29.72
11.47
190
180
170
160
150
140
130
120
110
100
90
80
70
60
50
40
30
20
ppm

+MS, 0.1-0.2min #5-9

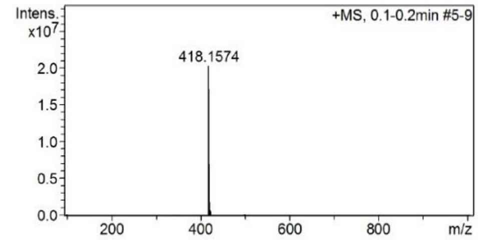

#	m/z	Res.	S/N	I	I %	FWHM
1	418.1574	13465	56984.1	20377596	100.0	0.0311

5

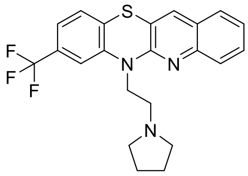

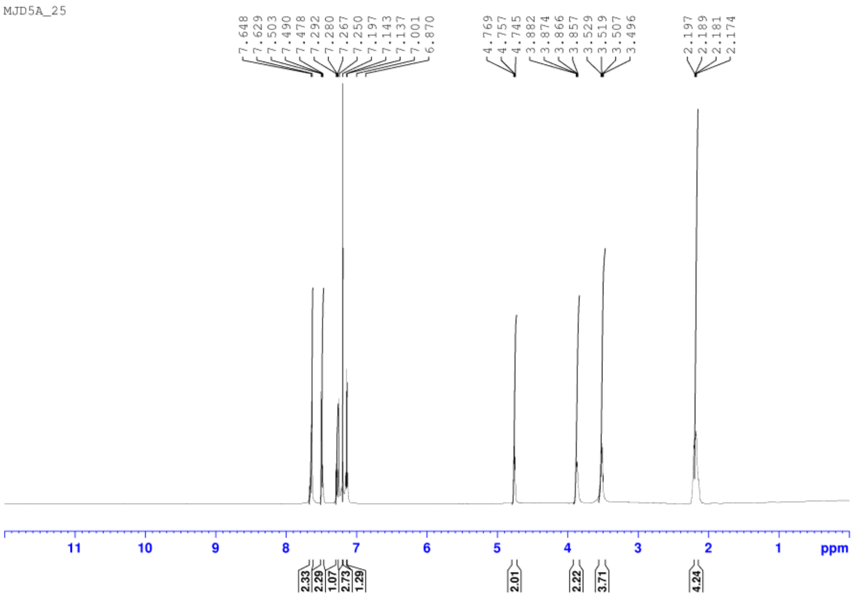

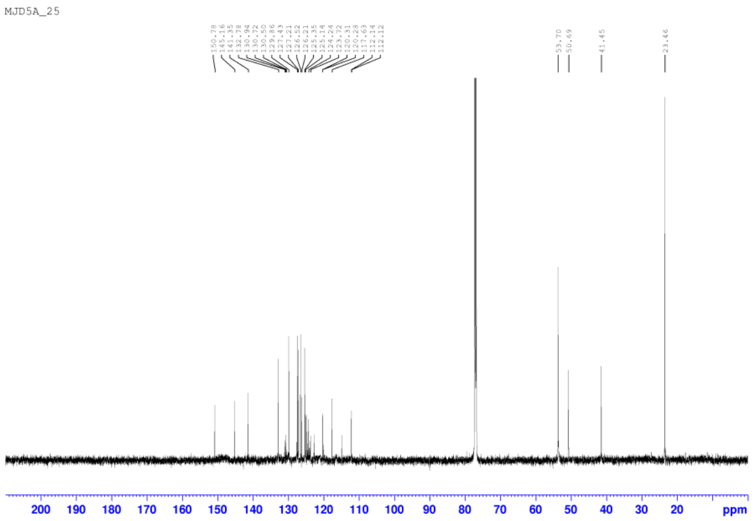

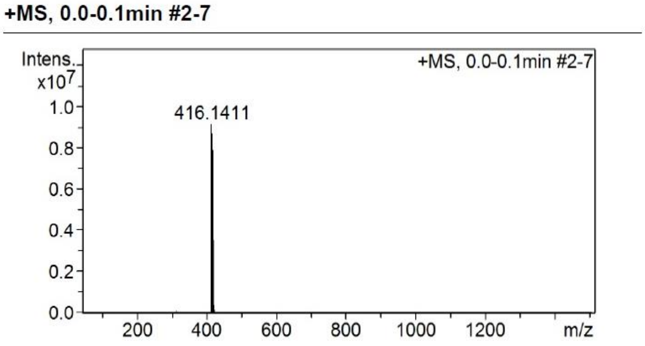

#	m/z	I	Res.
1	416.1411	9160687	6995
2	417.1429	8667596	18582

6

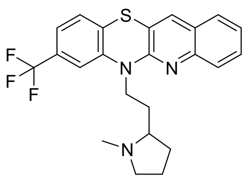

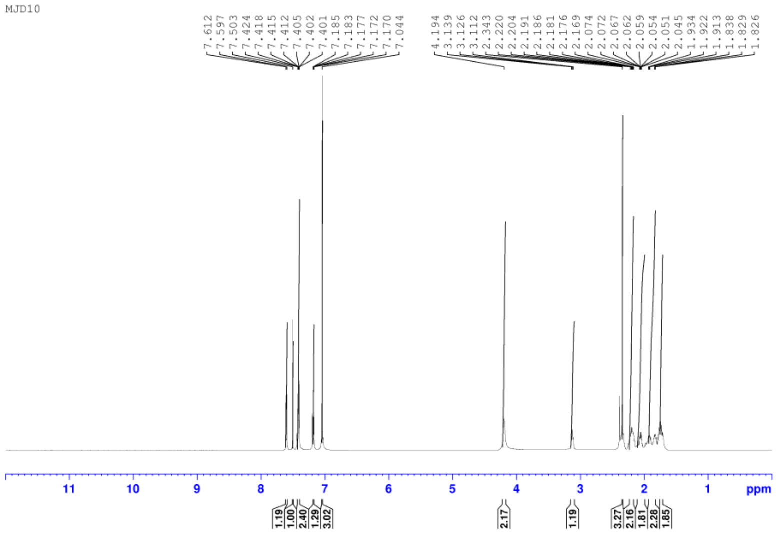

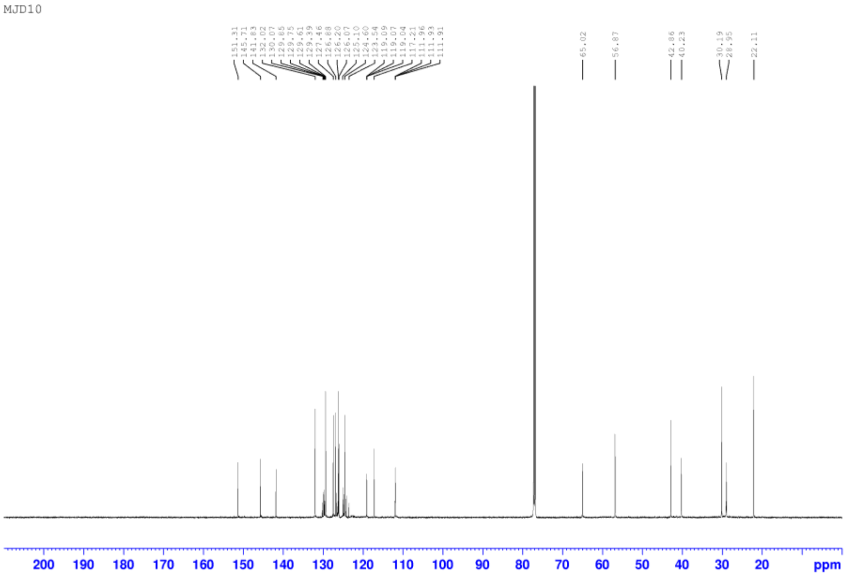

+MS, 0.1-0.2min #5-9

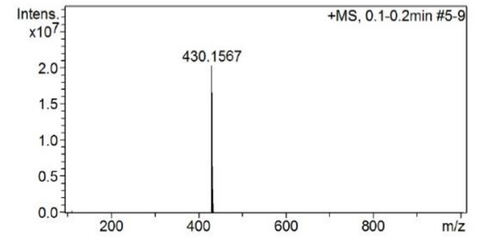

#	m/z	Res.	S/N	I	I %	FWHM
1	430.1567	16577	34818.3	20377596	100.0	0.0259
2	431.1602	44804	16601.8	9713004	47.7	0.0096

7

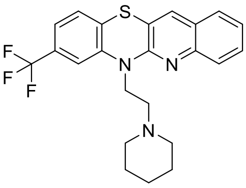

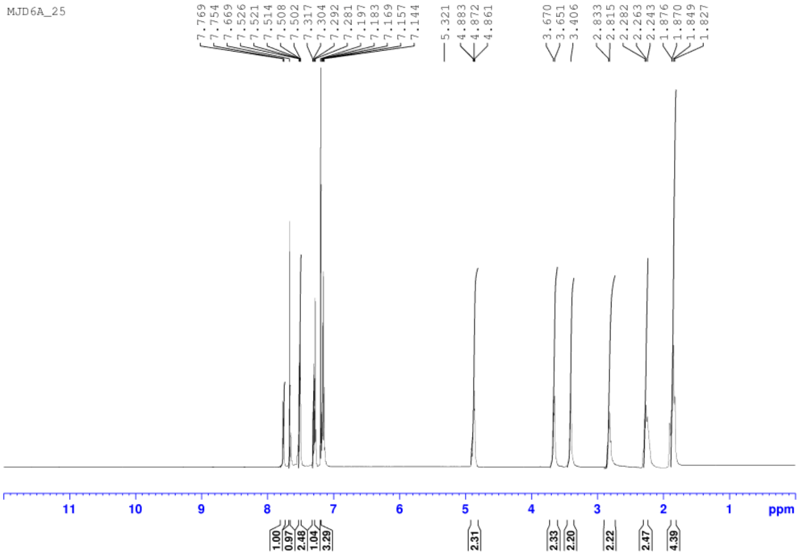

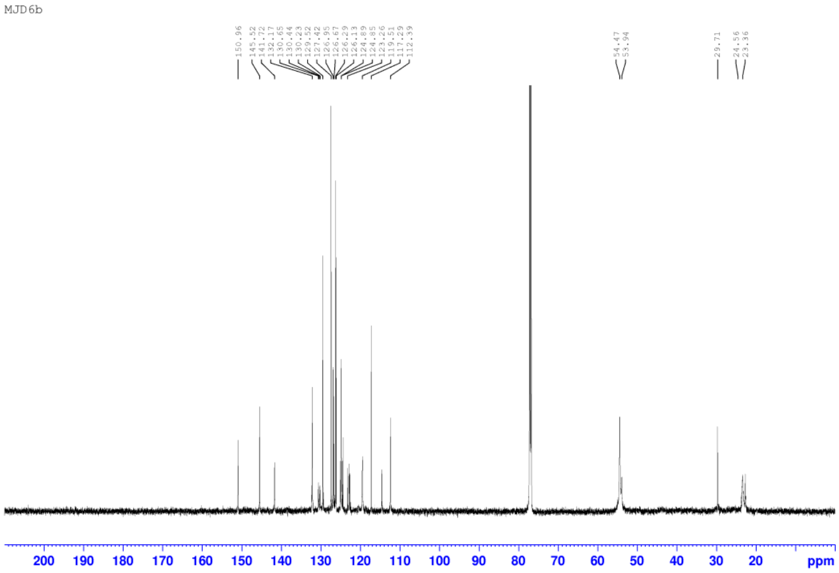

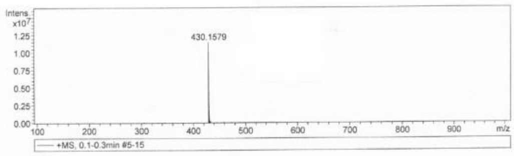

#	m/z	Res.	S/N	I	I %	FWHM
1	430.1579	44144	130473.7	11501014	100.0	0.0097
2	431.1609	37244	30878.2	2728462	23.7	0.0116

8

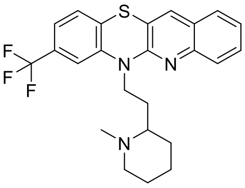
S
N
N
F
F
F
N

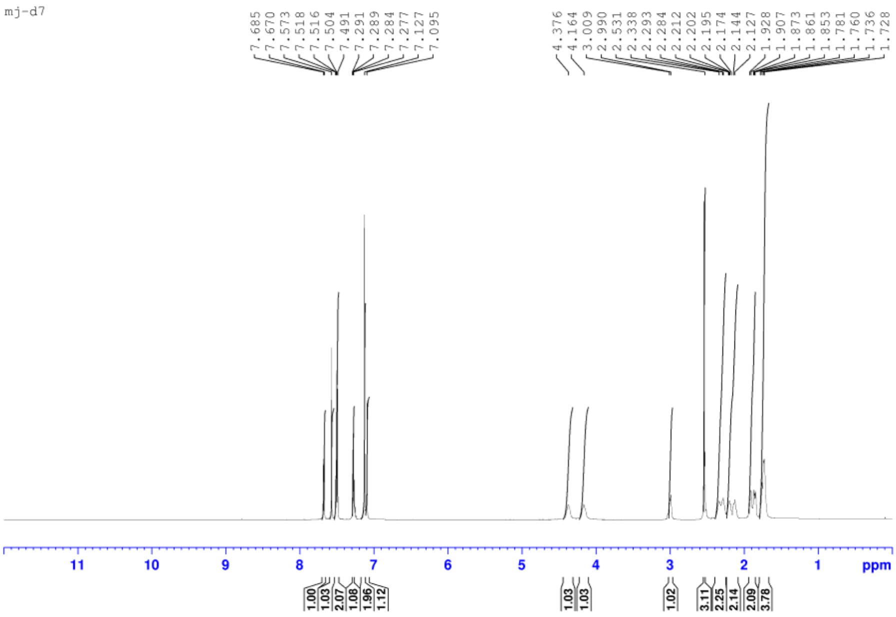
mj-d7
7.685
7.670
7.573
7.518
7.516
7.504
7.491
7.291
7.289
7.284
7.277
7.127
7.095
4.376
4.164
3.009
2.990
2.531
2.338
2.293
2.284
2.212
2.202
2.195
2.174
2.144
2.127
1.928
1.907
1.873
1.861
1.853
1.781
1.760
1.736
1.728
11 10 9 8 7 6 5 4 3 2 1 ppm
1.00
1.03
2.07
1.08
1.96
1.12
1.03
1.03
1.02
3.11
2.25
2.14
2.09
3.78

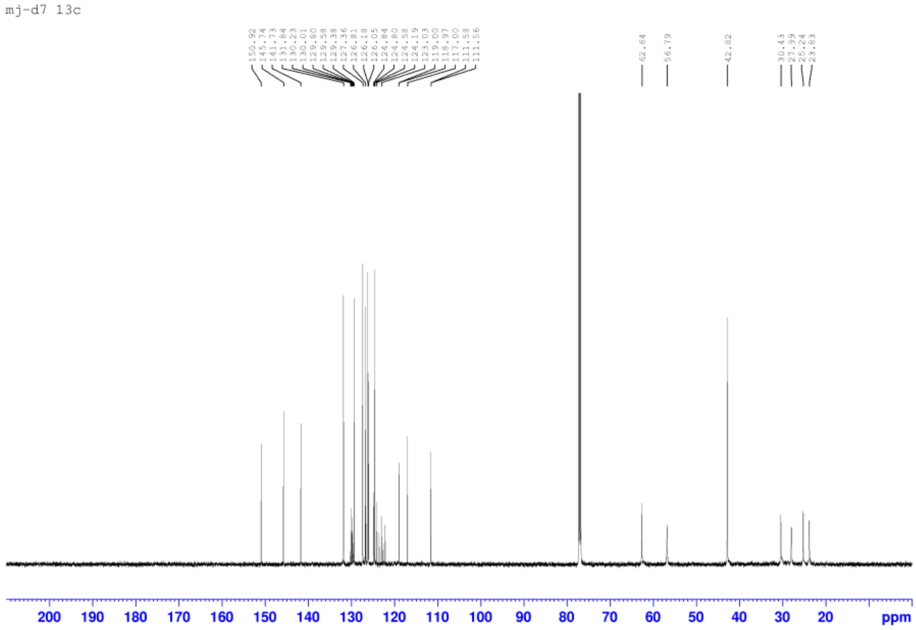
mj-d7 13c
150.92
145.74
141.73
131.84
130.23
130.01
129.80
129.58
129.38
127.36
126.81
126.18
126.05
124.84
124.80
124.58
124.19
123.03
119.00
118.97
117.00
111.58
111.56
62.64
56.79
42.82
30.43
27.99
25.24
23.83
200 190 180 170 160 150 140 130 120 110 100 90 80 70 60 50 40 30 20 ppm

+MS, 0.1-0.3min #3-15

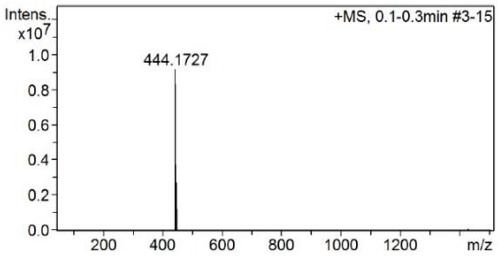

#	m/z	I	Res.
1	444.1727	9160702	6531
2	445.1743	9144140	16101

9

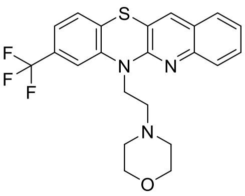

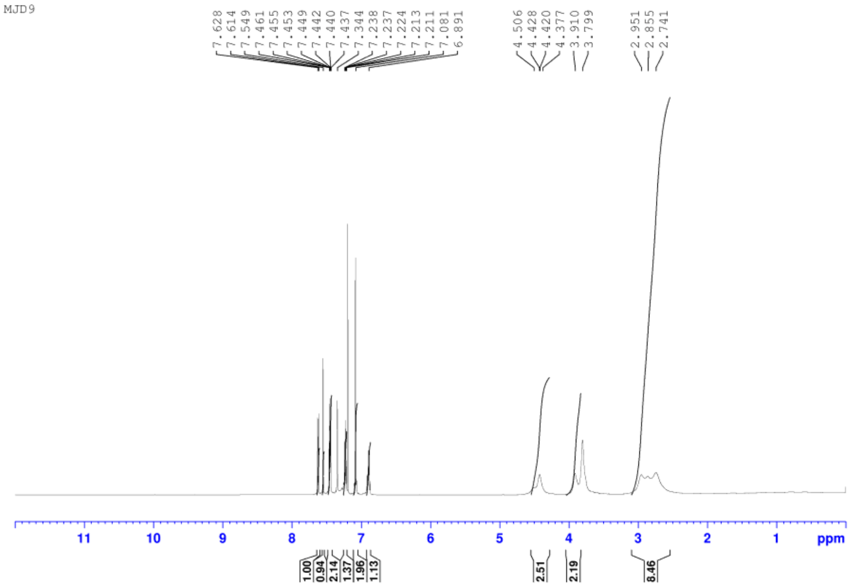

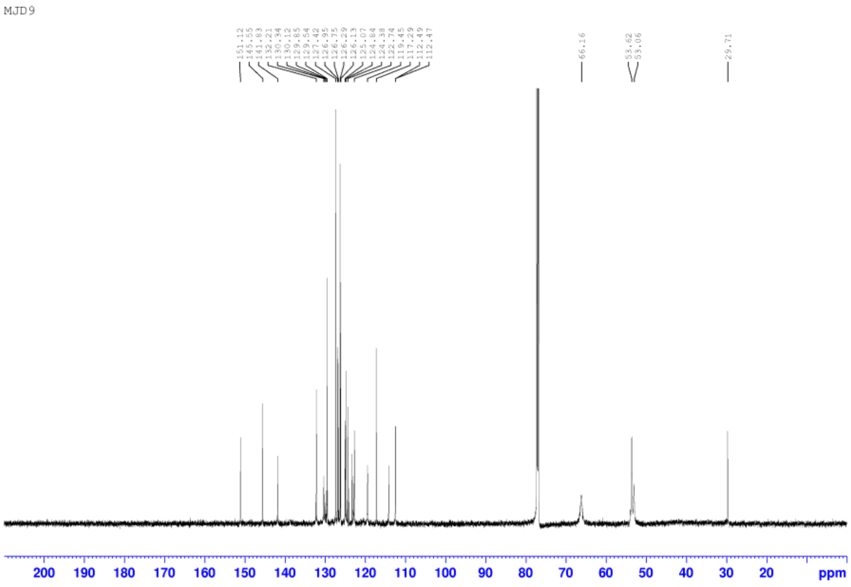

+MS, 0.1-0.3min #3-15

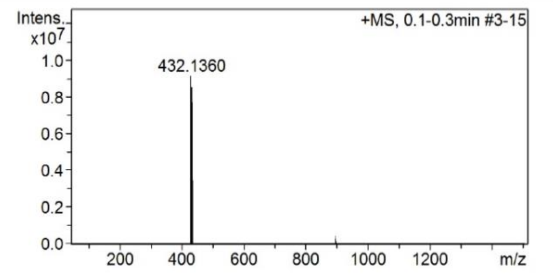

#	m/z	I	Res.
1	432.1360	9159443	7226
2	432.1360	9159443	7226

10

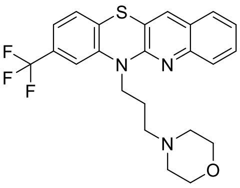
S
N
N
F
F
F
N
O

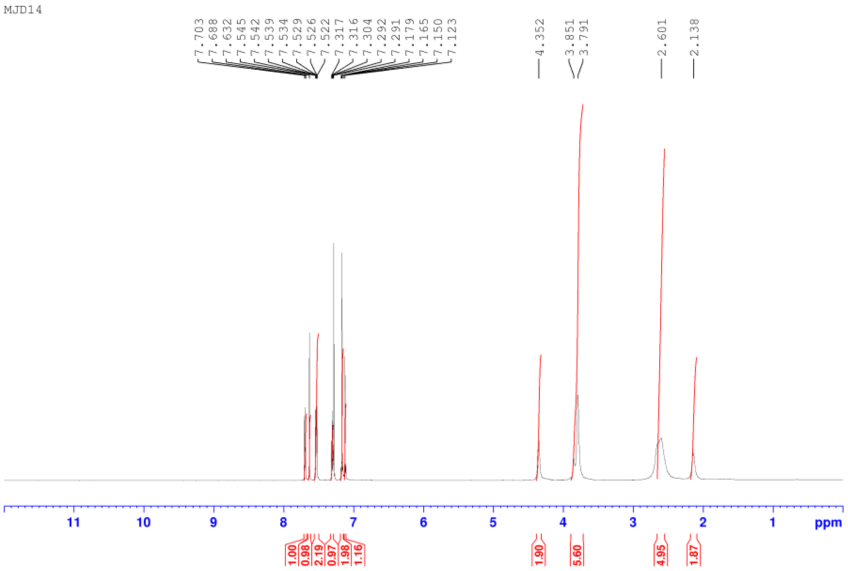
MJD14
7.703
7.688
7.632
7.545
7.542
7.539
7.534
7.529
7.526
7.522
7.317
7.316
7.304
7.292
7.291
7.179
7.165
7.150
7.123
4.352
3.851
3.791
2.601
2.138
11 10 9 8 7 6 5 4 3 2 1 ppm
1.00
0.98
2.19
0.97
1.98
1.16
1.90
5.60
4.95
1.87

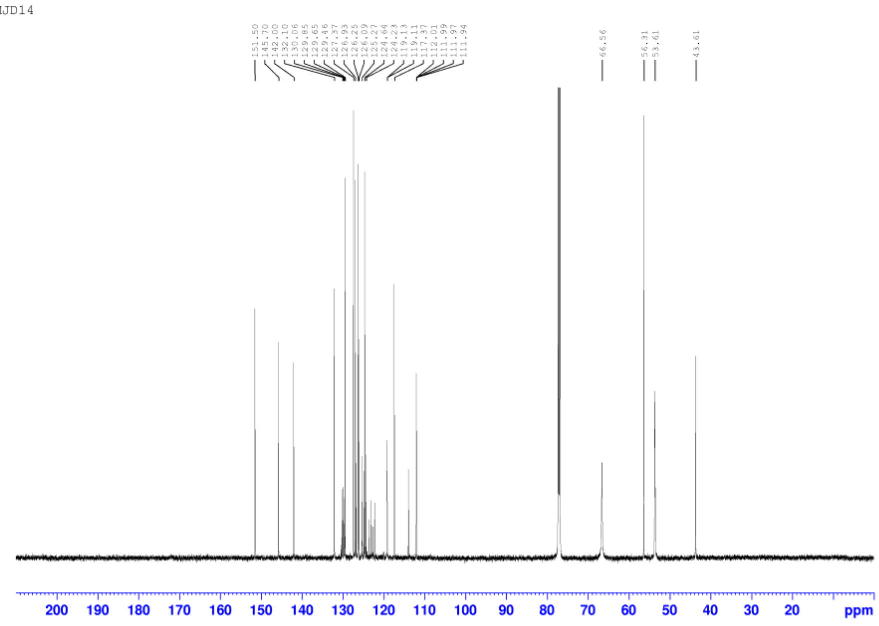
MJD14
151.50
145.70
142.00
132.10
130.06
129.85
129.65
129.46
127.37
126.93
126.25
126.09
125.27
124.64
124.23
119.13
119.11
117.37
112.01
111.99
111.97
111.94
66.56
56.31
53.61
43.61
200 190 180 170 160 150 140 130 120 110 100 90 80 70 60 50 40 30 20 ppm

+MS, 0.2min #11

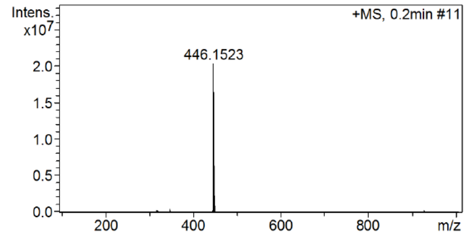

#	m/z	Res.	S/N	I	I %	FWHM
1	446.1523	8916	17002.4	20377592	100.0	0.0500
2	447.1554	42001	11965.2	14312074	70.2	0.0106

11

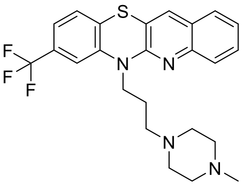

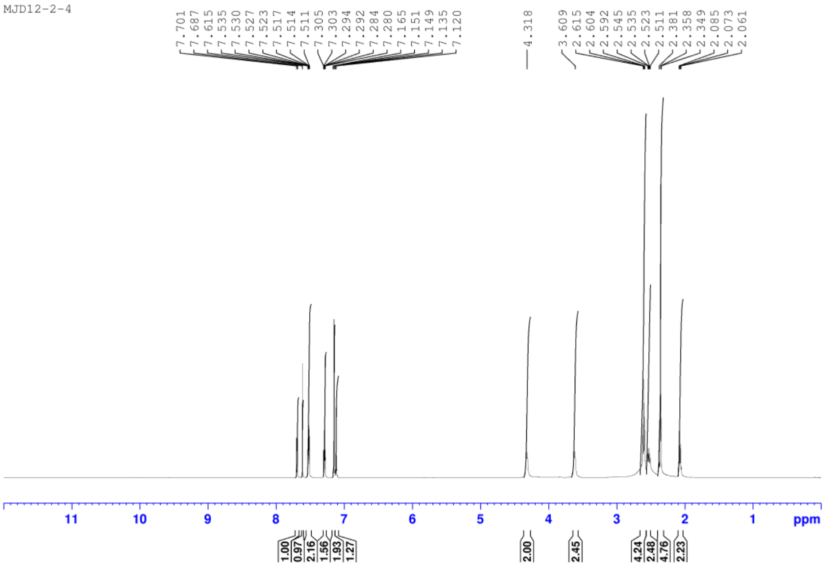

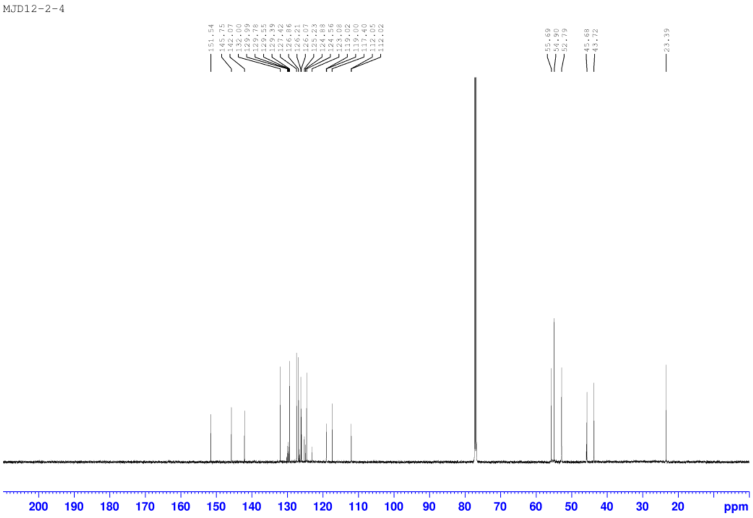

+MS, 0.1-0.2min #5-9

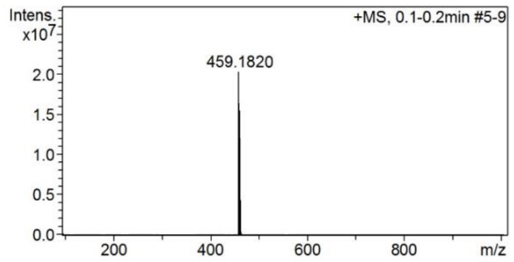

#	m/z	Res.	S/N	I	I %	FWHM
1	459.1820	11294	32191.0	20377596	100.0	0.0407
2	460.1874	46990	24564.3	15485573	76.0	0.0098

12

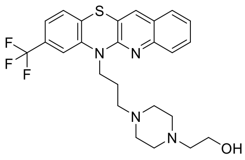

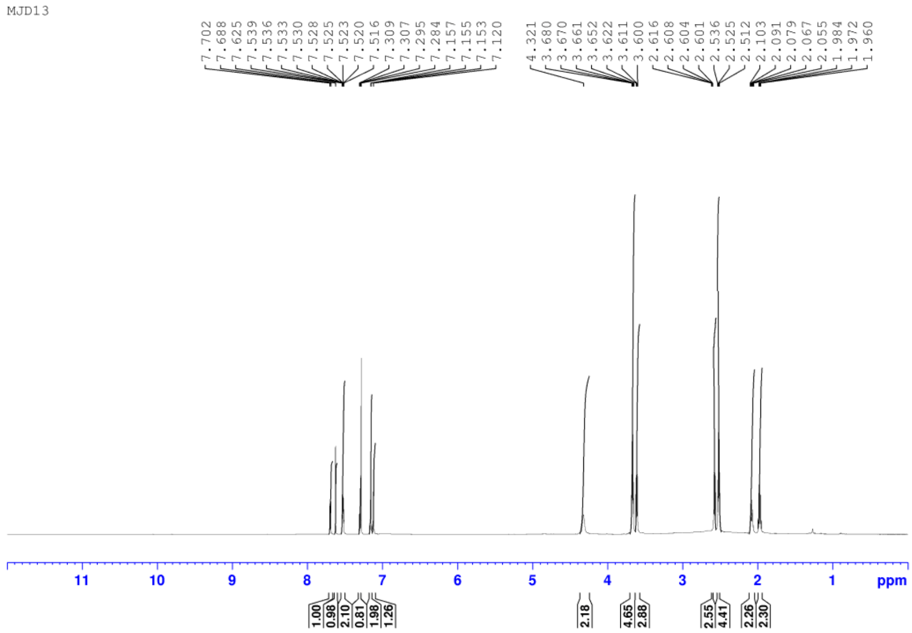

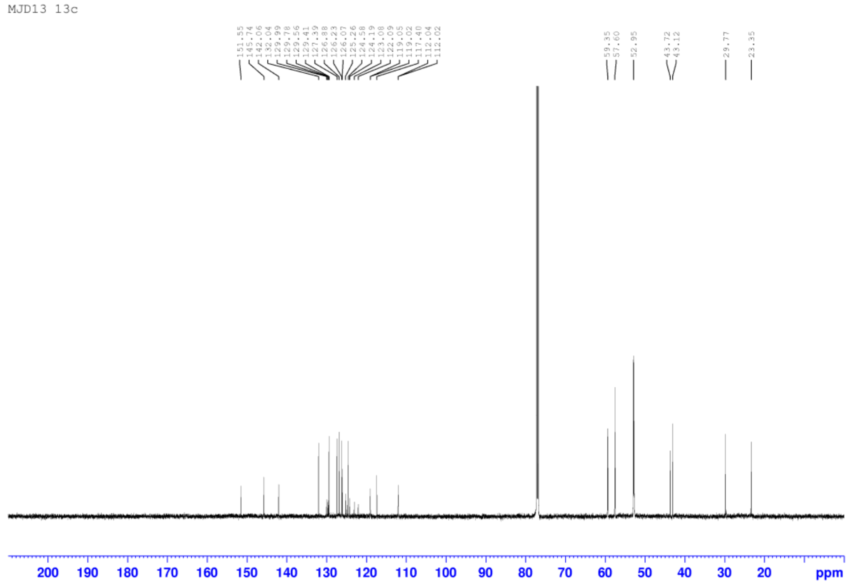

+MS, 0.2-0.2min #10-11

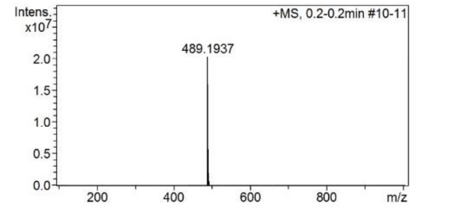

#	m/z	Res.	S/N	I	I %	FWHM
1	489.1937	12250	28574.1	20372552	100.0	0.0399
2	490.1972	46523	18360.0	13070959	64.2	0.0105

13

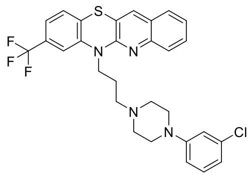

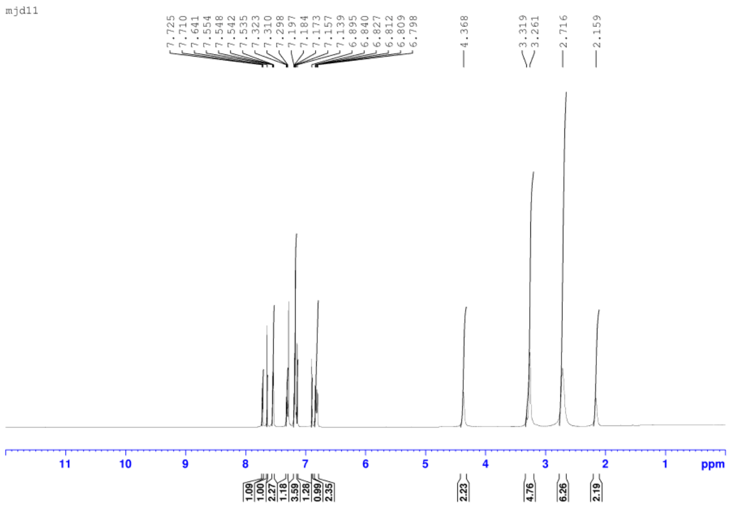

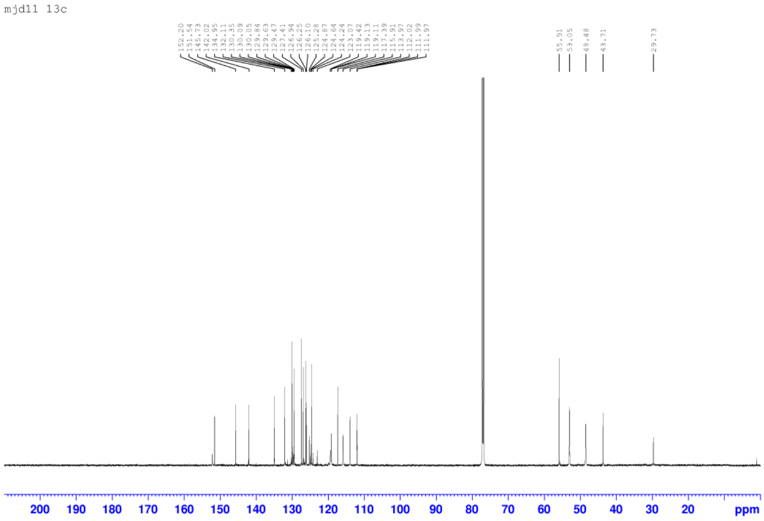

+MS, 0.3-0.3min #17-18

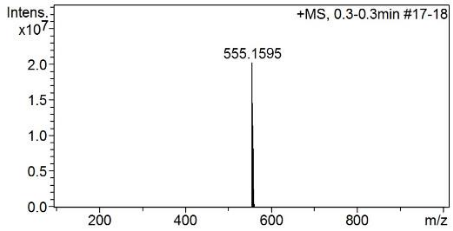

#	m/z	Res.	S/N	I	I %	FWHM
1	555.1595	17794	24957.9	20296260	100.0	0.0312
2	557.1580	46193	14129.5	11455775	56.4	0.0121
